# Marine gregarine genomes reveal the breadth of apicomplexan diversity with a partially conserved glideosome machinery

**DOI:** 10.1186/s12864-022-08700-8

**Published:** 2022-07-02

**Authors:** Julie Boisard, Evelyne Duvernois-Berthet, Linda Duval, Joseph Schrével, Laure Guillou, Amandine Labat, Sophie Le Panse, Gérard Prensier, Loïc Ponger, Isabelle Florent

**Affiliations:** 1 Département Adaptations du Vivant (AVIV), Molécules de Communication et Adaptation des Microorganismes (MCAM, UMR 7245 CNRS), Muséum National d’Histoire Naturelle, CNRS, CP 52, 57 rue Cuvier, 75231 Cedex 05 Paris, France; 2 Département Adaptations du Vivant (AVIV), Structure et instabilité des génomes (STRING UMR 7196 CNRS/INSERM U1154), Muséum National d’Histoire Naturelle, CNRS, INSERM, CP 26, 57 rue Cuvier, 75231 Cedex 05 Paris, France; 3 Present Address: Department of Biology, Lund University, Sölvegatan 35, 223 62 Lund, Sweden; 4 Département Adaptations du Vivant (AVIV), Physiologie Moléculaire et Adaptation (PhyMA UMR 7221 CNRS), Muséum national d’Histoire naturelle, CNRS, CP 32, 7 rue Cuvier, 75005 Paris, France; 5 CNRS, UMR7144 Adaptation et Diversité en Milieu Marin, Ecology of Marine Plankton (ECOMAP), Station Biologique de Roscoff SBR, Sorbonne Université, 29680 Roscoff, France; 6 Plateforme d’Imagerie Merimage, FR2424, Centre National de la Recherche Scientifique, Station Biologique de Roscoff, 29680 Roscoff, France; 7 Cell biology and Electron Microscopy Laboratory, François Rabelais University, 10 Boulevard Tonnellé, 3223 Cedex Tours, BP France

**Keywords:** Apicomplexa, Marine gregarine, Genome assembly, Comparative genomics, Gliding, Phylogeny

## Abstract

Our current view of the evolutionary history, coding and adaptive capacities of Apicomplexa, protozoan parasites of a wide range of metazoan, is currently strongly biased toward species infecting humans, as data on early diverging apicomplexan lineages infecting invertebrates is extremely limited. Here, we characterized the genome of the marine eugregarine *Porospora gigantea*, intestinal parasite of Lobsters, remarkable for the macroscopic size of its vegetative feeding forms (trophozoites) and its gliding speed, the fastest so far recorded for Apicomplexa. Two highly syntenic genomes named A and B were assembled. Similar in size (~ 9 Mb) and coding capacity (~ 5300 genes), A and B genomes are 10.8% divergent at the nucleotide level, corresponding to 16–38 My in divergent time. Orthogroup analysis across 25 (proto)Apicomplexa species, including *Gregarina niphandrodes*, showed that A and B are highly divergent from all other known apicomplexan species, revealing an unexpected breadth of diversity. Phylogenetically these two species branch sisters to Cephaloidophoroidea, and thus expand the known crustacean gregarine superfamily. The genomes were mined for genes encoding proteins necessary for gliding, a key feature of apicomplexans parasites, currently studied through the molecular model called glideosome. Sequence analysis shows that actin-related proteins and regulatory factors are strongly conserved within apicomplexans. In contrast, the predicted protein sequences of core glideosome proteins and adhesion proteins are highly variable among apicomplexan lineages, especially in gregarines. These results confirm the importance of studying gregarines to widen our biological and evolutionary view of apicomplexan species diversity, and to deepen our understanding of the molecular bases of key functions such as gliding, well known to allow access to the intracellular parasitic lifestyle in Apicomplexa.

**Supplementary Information:**

The online version contains supplementary material available at 10.1186/s12864-022-08700-8.

## Background

Apicomplexans are unicellular eukaryotic microorganisms that have evolved towards endobiotic symbionts or parasites. The Apicomplexa include about 350 genera [1] for 6000 documented species. Some species are extremely pathogenic such as *Plasmodium* spp., *Toxoplasma gondii* and *Cryptosporidium* spp., responsible for malaria, toxoplasmosis and cryptosporidiosis, respectively. Current knowledge of apicomplexan genomes is based on sequence data from a dozen genera, and more precisely, the genera which include highly pathogenic species to humans [2]. Consequently, our view of the Apicomplexa genomes is highly skewed towards intracellular parasites of vertebrates, notably Coccidia, Hemosporidia and *Cryptosporidium* (see references in Table S1). By comparison, the gregarines, of which there are at least 1770 species [3], have hardly been explored at an omic level [4]. Gregarines were identified as the most abundant and widely reported apicomplexan in a recent environmental study [5]. However, as they have low pathogenicity and are non-cultivable in the laboratory, they have attracted less interest.

Overlooking the gregarines risks leaving part of the evolutionary history of Apicomplexa unexplored, because they represent early diverging lineages as well as displaying a diversity of specific adaptive traits. For instance, gregarines are mostly extracellular, infecting a wide diversity of marine and terrestrial non-vertebrate hosts [6, 7]. At this time, available genomic data are very limited to terrestrial gregarines, such as partial data on *Ascogregarina taiwanensis,* an intestinal parasite of the tiger mosquito *Aedes albopictus* [8], and the draft genome of *Gregarina niphandrodes,* an intestinal parasite of the mealworm *Tenebrio molitor* (unpublished, available in CryptoDB [9]). Transcriptomic studies on trophozoite (feeding) stages of terrestrial and marine gregarine species have recently provided important insights [10–13], especially about organellar genomes and metabolic pathways. These developmental stage-dependent data, however, do not provide a complete picture of the genetic landscape of gregarines, nor can they provide information on their genome structure.

To study the gregarine genomes, we focused on the marine eugregarine *Porospora gigantea* (Van Beneden, 1869) Schneider, 1875, which is an intestinal parasite of the lobster *Homarus gammarus.* First described in 1869, E. Van Beneden named the organism *Gregarina gigantea* in reference to the “gigantic” size (up to 16,000 μm) of the trophozoite stages, being visible to the naked eye [14]. Van Beneden reported that “cyst” forms of this parasite accumulated within the chitinous folds of the lobster rectum, the “rectal ampulla”. Schneider went on to show that these cysts enclosed thousands of “gymnospores” or “heliospores”, corresponding to spherical groups of very tiny zoites radiating from a central, optically void mass, and renamed the species *Porospora gigantea* (Van Beneden, 1869) Schneider, 1875 [15]. Biological material for genomic studies is particularly difficult to gather from non-cultivable microorganisms, so we took advantage of the existence of these well-described structures [16–19], knowing that each cyst contains several thousand “gymnospores”, each composed of hundreds of zoites, involving the natural amplification of its genomic material. Cysts indeed proved to be a remarkable natural source of genomic DNA. Gliding is a characteristic apicomplexan movement that also happens to be essential for the invasion and egress of host cells, and thus for the intracellular parasitic lifestyle [20–24]. *P. gigantea* trophozoites are known to glide at rates of up to 60 μm/s [25], so are prime candidates in which to study the mechanism of gliding motility. Currently about 40 proteins, identified mainly in *T. gondii* and *Plasmodium falciparum*, compose the glideosome, a commonly accepted structural model of this apicomplexan motor complex (see Frénal et al., 2017 [26] for review).

In this study, we report the first draft genome of *P. gigantea*. Remarkably, not one but two related genomes have been assembled. We present their main features and predicted proteomes and compare them to other available apicomplexan genomes, revealing an unexpected diversity. We investigated their position within Apicomplexa and among the major subgroups of gregarines through a phylogenomic analysis. We also examined their position within the crustacean gregarines according to 18S ribosomal gene sequences. Finally, a comparative study was performed to gain insight into the conservation of gliding proteins for these gregarines, the currently fastest moving extracellular Apicomplexa.

## Results

### Phenotypic characterization

**Fig. 1:**
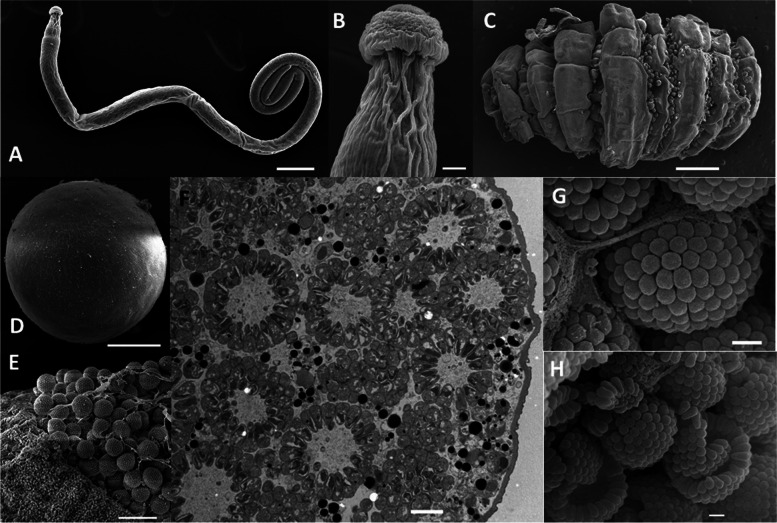
Morphological characterization of *Porospora* cf. *gigantea.* **A**. Trophozoite stage (Tropho #8, Lobster #12) (scale bar = 100 μm). **B**. Zoom on A, showing trophozoite epimerite (scale bar = 10 μm). **C**. Rectal ampulla showing cysts in folds (Lobster #4) (scale bar = 1 mm). **D**. Isolated cyst (Cyst #4, Lobster #12) (scale bar = 50 μm). **E**. Broken cyst packed with gymnospores (Lobster #4) (scale = 10 μm). **F**. Section across a cyst illustrating radial arrangement of zoites in gymnospores (JS449 = Lobster #35) (scale bar = 2 μm). **G**., **H**. Zoom on intact and broken gymnospores showing zoites (Lobster #4) (scale = 1 μm). All images are scanning electronic micrographs except F which is a transmission electronic micrograph. See also Fig. S1, Tables S2, S3, S4, S5 and S6

Specimens of the lobster *Homarus gammarus*, the type host species for *Porospora gigantea*, were collected either from the sea in Roscoff bay (France) or from commercial lobster tanks in Roscoff (Fig. 1, Table S2). A total of 35 lobsters (9 from the wild and 26 from captivity) were dissected and infection with *P. gigantea* was quantified (Fig. 1, Fig. S1). Overall, infection levels were significantly higher in lobsters freshly caught from the sea (prevalence of 100%, high parasitic loads) than in lobsters that had been held in captivity in lobster tanks (prevalence < 62%, low parasitic loads, see Table S2), a similar result to that reported by Van Beneden (1869) [14]. The morphology of cysts, gymnospores, zoites and trophozoites was imaged and measured (Fig. 1, Tables S3, S4 and S5). Cysts were mostly spherical but some were ovoid, with diameters ranging from ~ 104 μm to ~ 252 μm (mean ± standard deviation, 151.1 ± 45.3 μm, *n* = 97), and they enclosed thousands of gymnospores, that were also mostly spherical, with diameters from less than 5 μm to almost 7 μm (5.63 ± 0.69 μm, *n* = 265). These gymnospores were indeed composed of radially arranged zoites forming a monolayer with an optically void center. Observation of broken gymnospores by scanning electron microscopy made it possible to measure the length of the constituent zoites (1.04 ± 0.16 μm, *n* = 105) and their apical width (0.630 ± 0.129 μm, *n* = 176). Trophozoites were very thin and long, up to 2585 μm for a mean width of 41.8 ± 10.4 μm (*n* = 104). As previously described, the posterior of the trophozoite was slightly thinner, ~ 30 μm. The whole trophozoite surface was covered by longitudinal epicytic folds (Fig. S1.B) that are thought to be necessary for eugregarine gliding [27]. The sum of these morphological observations all accord with the species being *P. gigantea* from the type host *H. gammarus* [6, 14, 15].

Gliding of isolated trophozoites was filmed. The dynamic recordings confirm that trophozoites moved uni-directionally, with the protomerite forwards, in either straight or curved lines depending on the individuals observed, with the whole body (deutomerite) following the same path as the apical protomerite (Film S1). The speed of trophozoite displacement was estimated to be ~ 60 μm/sec, as initially observed by King and Sleep (2005) [25], but was faster than 100 μm/sec in some recordings (Table S6). No syzygy was observed. A few solitary encysted trophozoites were observed, supporting the observations of Léger and Duboscq (1909) [28], who considered that encysted gymnospores correspond to a schizogonic rather than gamogonic phase of *Porospora* development. This hypothesis is still open since the gamogonic phase with male and female gametes and the fertilization process are not yet documented in the life cycle of *P. gigantea* [6].

### Two highly related genomes

Four biological samples were sequenced and analyzed independently, and then assembled together (Fig. S2.A). The raw assembly produced 214,938 contigs (99.6 Mb) among which were 13,656 contigs longer than 1 kb (47.9 Mb). The scaffolds obtained were cleaned by removing contaminants such as bacterial, fungal and host sequences (Fig. S2.B), resulting in a raw assembly of 1719 contigs covering 18 Mb.

The analysis of contig coverage for each individual library revealed a bimodal distribution suggesting a mixture of genomes in differing proportions depending on the biological sample (Fig. S3). More precisely, while only one set of contigs displayed a significant coverage for the lobster tank parasite sample (JS-470, peak around 250×), the three other parasite samples from freshly captured hosts (JS-482, JS-488, JS-489) showed two distinct sets of scaffolds with similar size (~ 9 Mb) and different coverage values. The difference in coverage was used to split the whole assembled contigs into two sets that were named A for the set of contigs present in all four samples, and B for the set present only in the three lobsters freshly captured in the wild (Fig. S2.C). The percentages of genomes A and B in each biological DNA sample was estimated (Fig. S3) as 100% A for JS-470, 63.2% A and 36.8% B for JS-482, 70.5% A and 29.5% B for JS-488, and 62.4% A and 37.6% B for JS-489, based on medium coverage levels. Genome A maps to 787 contigs for a total of 8.8 Mb, whereas genome B maps to 933 contigs for a total of 9.0 Mb. Contigs from the two genomes can be aligned with each other over 7.7 Mb, with a percentage of divergence around 10.8% at the nucleotide level.

To summarize, these two genomes have a similar size (~ 9 Mb) and are syntenic with nevertheless 10.8% of divergence. These highly related genomes have been named A and B and are associated to the species name *P.* cf. *gigantea* (Fig. S2).

### Genome features

#### Two genomes with similar coding capacities

A total of 10,631 putative genes were predicted from the raw assembly (17,930 alternative splicings), which were split into two sets of similar size: 5270 genes in genome A (8895 alternative splicings) and 5361 genes (9035 alternative splicings) in genome B (Table 1, Fig. S2). The completeness of both A and B genomes was assessed by using BUSCO software [29] on the Apicomplexa geneset (*n* = 446). Genomes A and B respectively showed completeness scores of 70% (*n* = 312) and 67.7% (*n* = 302) (Fig. S4).

**Table 1:** Metrics of the genomes of *P.* cf. *gigantea* and a selection of 6 reference species. * by considering only genes with intron(s). See also Figs. S2, S3 and S5

species	*P.* cf. *gigantea*		*G. niphandrodes*	*C. parvum*	*T. gondii*	*P. falciparum*	*C. velia*	*V. brassicaformis*
strain	A	B	na	IowaII	ME49	3D7	CCMP2878	CCMP3155
nb of contigs/chromosomes	787	934	355	8	435	14	5470	1006
total length of assembly (bp)	8,806,768	9,049,943	13,873,624	9,102,324	63,472,444	23,292,622	192,006,978	72,475,329
mean length contigs/chromosomes (bp)	11,190.3	9689.45	39,080.63	1,137,790.5	145,913.66	1,663,758.71	35,101.82	72,043.07
GC content (%)	54.3	54.3	53.8	30.2	52.4	19.3	49.1	58.1
nb of protein coding genes	5270	5361	6606	4020	8862	5602	30,604	23,412
mean length of coding genes (bp)	1438.2	1450.3	1392.6	1865.0	5602.9	2488.6	4507.6	2704.7
nb of tRNA	14	14	231	45	150	45	0	0
nb of rRNA	27	25	0	5	420	28	0	0
nb of gene with intron(s)	2957	2981	2390	575	6801	3010	21,895	22,163
median length of the introns (bp)	28 [27–30]	28 [27–30]	95 [56–145]	65 [51–91]	467 [322–632]	140 [110–184]	372 [273–520]	81 [70–98]
mode of intron length (bp)	28	28	37	44	55	121	320	74
mean nb of introns per gene*	1.8	1.8	1.4	1.8	5.9	2.9	5.4	7.9
non-coding DNA (%)	16	16	37	24	68	47	74	50

The number of A and B orthologues was investigated. The predicted proteins of *P.* cf. *gigantea* A and B were split into 5656 orthogroups including 4443 groups (88%) which had at least one orthologous gene for both A and B. This percentage of common orthogroups between genomes A and B is higher than that observed between *Plasmodium falciparum* and *Plasmodium berghei* (70%), thought to have diverged around 33 Mya ago (TimeTree [30]), but similar to that observed between *P. falciparum* and *Plasmodium reichenowi* (86%, 3.3–7.7 Mya, TimeTree).

**Fig. 2:**
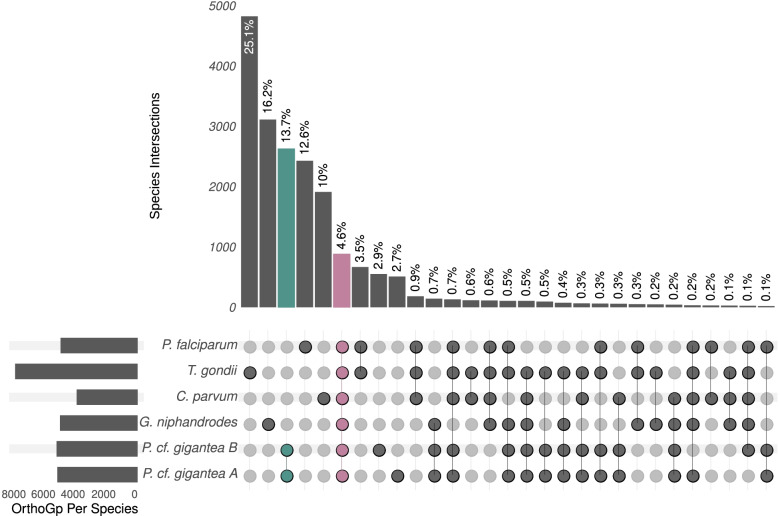
Shared apicomplexan proteins. Distribution of the orthogroups among *P.* cf. *gigantea* A and B and 4 species of apicomplexans: the gregarine *G. niphandrodes*, the cryptosporidian *C. parvum,* the coccidian *T. gondii* and the hematozoan *P. falciparum*. Orthogroups only shared by *P.* cf. *gigantea* A and B are highlighted in green, whereas orthogroups shared by all species are highlighted in pink. Only bars with more than 20 orthogroups are shown. See also Table S1

The percentages of shared orthogroups between *P.* cf. *gigantea* genomes and each of the reference apicomplexan species are similar (*Cryptosporidium parvum*, 18%; *G. niphandrodes*, 17%; *P. falciparum*, 14%; *T. gondii*, 14%) despite the differences in divergence, but it is higher than the percentages observed with chromerid species (*Chromera velia*, 8%; *Vitrella brassicaformis,* 10%). We can deduce from these results that the *P.* cf. *gigantea* genomes do not share significantly more orthogroups with *G. niphandrodes*, the only other available gregarine genome, than with any other apicomplexan (Fig. 2).

#### Two gene-dense genomes with small introns

The proportion of coding sequences in A and B genomes is 84%, which is particularly high compared to other reference species (with values ranging from 25 to 76%; Table 1). The genomic compaction of non-coding DNA in genomes A and B can be explained by the shortness of most introns (Fig. S5). A specific class of introns with lengths around 25–30 bp (mode at 28 bp) represents 71–72% of the introns. The donor and acceptor sites of these small introns have specific consensus patterns (Fig. S5) which are different from other *Porospora* introns. Specifically, these introns exhibit a strongly conserved adenine located 6 bp upstream of the 3′ acceptor site which could represent the intron branch point, as observed for the small introns (20 bp) in *B. microti* [31].

#### Loss of organellar genomes

Recent studies suggest that organellar genomes are lost in most gregarines [10, 32]. A precise protocol was set up to identify putative contigs associated with organellar genomes in *P. gigantea*. All the assembled contigs (assigned to *P. gigantea* or not) were searched for regions similar to known organellar genomes. A sensitive protocol based on TBLASTX identified 108 putative regions that were aligned to the NCBI NR library. 102 regions were discarded as bacterial contamination. The 4 contigs corresponding to the remaining 6 regions with at least one significant hit against an eukaryotic sequence were manually curated. Two contigs were assigned to host-derived contaminants whereas the two other long contigs (L = 24,892 and L = 33,594) corresponded to *P. gigantea* nuclear genome. Thus, our analyses did not reveal any putative contigs compatible with mitochondrial or apicoplastic genomes.

### Evolutionary histories of *P.* cf. *gigantea*

#### Genomes A and B diverged several million years ago

We estimated the putative divergence time of A and B genomes by using the divergence between *P. falciparum* and *P. reichenowi* as a calibration point. The synonymous divergence (dS) was calculated for 1003 quartets of orthologous genes. The mean dS value observed between *P. falciparum* and *P. reichenowi* orthologues was 0.0959, similar to that calculated by Neafsey et al. [33] (0.068 substitutions per site) or Reid et al. [34] (0.086–0.11 per site). We assumed that these *Plasmodium* species diverged between 3.3 and 7.7 Mya (TimeTree). The mean dS value observed between the same orthologues in both *P.* cf. *gigantea* genomes was about 0.4295 substitutions per site. Assuming similar substitution rates in gregarines and *Plasmodium* species, we dated the split between genomes A and B to have occurred between 15.5 Mya and 37.7 Mya. This order of magnitude is similar to the estimation of when the basal splits of the mammal *Plasmodium* [35] (12.8 Mya) or all *Plasmodium* [36] (21.0–29.3 Mya) occurred, but is significantly later than the emergence of Nephropidae (lobster family) around 180 Mya [37, 38].

#### Expanded superfamily of crustacean gregarines

**Fig. 3:**
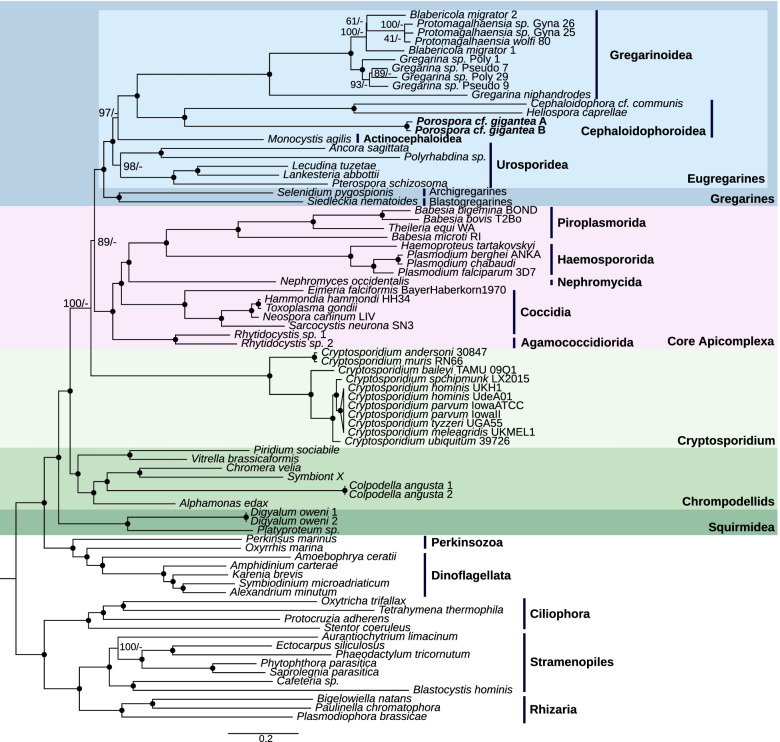
Phylogeny of Apicomplexa. Maximum likelihood phylogeny of apicomplexans as retrieved from a 312 proteins dataset, merged from two previously published datasets [10, 11, 13]. Final concatenated alignment comprised 93,936 sites from 80 species. Bootstrap support values (*n* = 1000) followed by MrBayes posterior probabilities are shown on the branches. Black spots indicate 100/1 supports. *Porospora* cf. *gigantea* A and B sequenced in this study are bolded. See also Figs. S7 and S8

To assess the position of *P.* cf. *gigantea* A and B within Apicomplexa, we constructed a genome-wide phylogeny based on 312 concatenated proteins from the datasets published by Salomaki et al., 2021 [13] and all recently published transcriptomic data from gregarines [10, 11, 13] (Fig. 3). This phylogeny grouped *P.* cf. *gigantea* A and B into one clade, placed as a sister group of other crustacean gregarines (*Cephaloidophora communis*, *Heliospora caprellae*), although having shorter branch lengths. In agreement to Salomaki et al. (2021) [13] *Cryptosporidium* species remain at the base of A + G (Apicomplexa + gregarines), using a LG + C60 + G + F model in maximum likelihood phylogenomic analyses. However, the bayesian analysis using classical partitioned model LG + G + F is in favor of a A + C topology (Apicomplexans + *Cryptosporidium*) (average standard deviation of split frequencies = 0.020977). More sampling of *Cryptosporidium* relatives is required to address the apicomplexan topology issue.

The sequences of 18S small subunit ribosomal DNA, for which the largest taxonomic sampling for gregarines is available in databases, was also used to position *P.* cf. *gigantea* within the crustacean gregarines. Using a combination of amplifications with specific primers (initially based on Simdyanov et al. (2015) [39] and Schrével et al. (2016) [40] then partly redesigned (Fig. S6, Table S7)) and in silico clustering, we were able to fully reconstruct complete ribosomal loci covering 18S-ITS1–5.8S-ITS2-28S (5977 bp) for both A and B genomes. Thirty polymorphic positions were found between A and B, only one within the 18S sequence, and 29 within the 28S sequence (Fig. S6). Two phylogenetic studies were performed, one excluding environmental sequences (Fig. S7), the other including them (Fig. S8). Most environmental sequences are derived from marine sediments from a wide range of habitats but only two sequences are from the North Atlantic where European and American lobsters live.

Congruent with the concatenated phylogeny (Fig. 3), both 18S phylogenies assigned *P.* cf. *gigantea* A and B to their own clade, placed as a sister group to all other crustacean gregarines (*Cephaloidophora*, *Heliospora*, *Thiriotia*, and *Ganymedes* species), as established in Rueckert et al. (2011) [41] (Figs. S7 and S8). Five main clades constituting the superfamily Cephaloidophoroidea were retrieved. The four clades previously outlined [41], redenominated as Ganymedidae, Cephalodophoridae, Thiriotiidae (as proposed by Desportes and Schrével (2013) [6]), and Uradiophoridae, had at their base the clade Porosporidae. Historically defined as the family gathering *Porospora* and *Nematopsis* genera [6], this clade is constituted of the two sequences of *P.* cf. *gigantea.* A new putative clade was formed by the five sequences from a Slovenian karst spring published by Mulec and Summers Engel (2019) [42] (Fig. S8), and it is very well supported to be a sister group to four of the crustacean gregarine families, while the family Porosporidae retains its position as a sister group to all these other clades.

### Partially conserved glideosome machinery

**Fig. 4:**
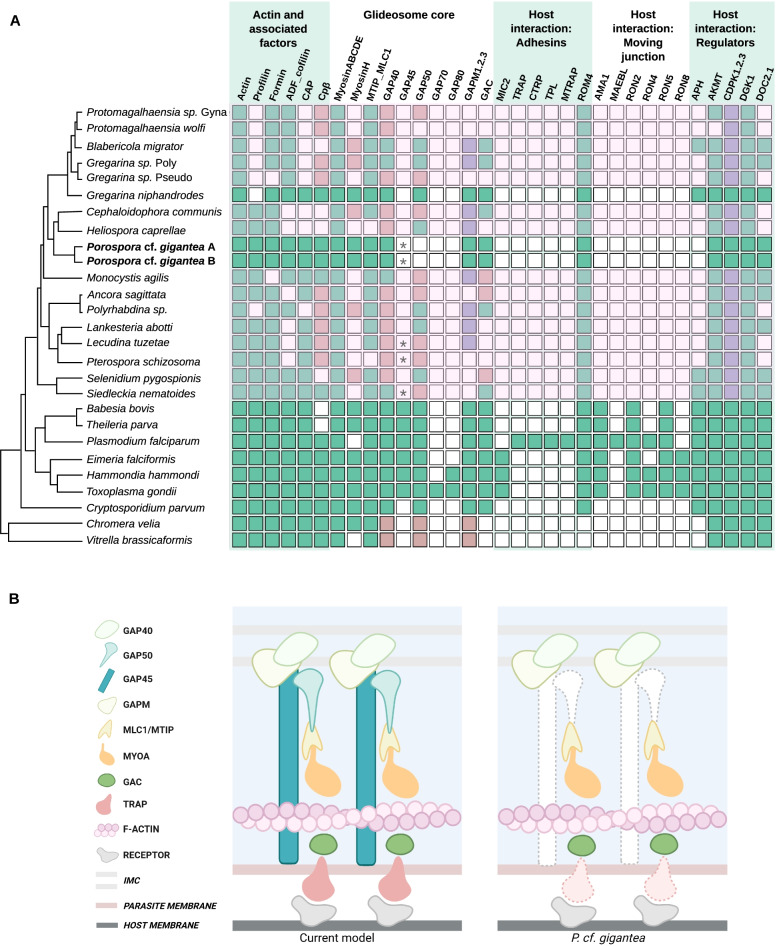
Comparative analysis of glideosome components. **A**. Table of presence/absence of genes encoding glideosome proteins, distributed into functional groups. Glideosome components have been described mainly in *T. gondii* and *P. falciparum*. Protein sequences were searched for in the genomes of both *Porospora* and a selection of representative species as well as in available gregarine transcriptomes. Green indicates the presence, while white indicates the absence of an orthologous protein-encoding sequence. Light red refers to cases where only partial sequences have been retrieved. Violet indicates the presence of at least one protein in multigenic family proteins. * refers to the GAP45 3′ short conserved domain found in some gregarines species. All *P.* cf. *gigantea* orthologous proteins are detailed in Table S8. **B**. Schematic comparison of the canonical model of the glideosome and the elements found in *P.* cf. *gigantea* A and B. Missing proteins are shown with dotted lines

We conducted an inventory of the presence or absence of genes encoding proteins involved in the gliding motility based on the molecular description of the so-called glideosome machinery, grouped according to their function as established by Frénal et al. (2017) [26] (Fig. 4A, all orthologues for *P.* cf. *gigantea* are detailed in Table S8). Genes for these *T. gondii* and *P. falciparum* reference proteins were searched for in both *P.* cf. *gigantea* genomes and in the genomes of a selection of representative species, as well as the recently published gregarine transcriptomes [10, 11, 13].

#### Actin and associated factors

Actin in apicomplexans is characterized by a globular monomeric form (G-actin) which polymerizes as needed into short unstable filaments (F-actin) [43] using various regulators such as profilin [44–46], ADF cofilin [47], formin [48–50], cyclase-associated proteins (CAP) [51] and F-actin capping protein Cpβ [52]. The inactivation of actin or its associated regulators compromises motility and host cell invasion and egress, although motility may persist in an altered form for a few days, perhaps through alternative mechanisms [26, 53–55]. Overall, these proteins are well conserved among Apicomplexa. However, profilin appears to be absent in insect-infecting Gregarinoridea; CAP and Cpβ also seem to be poorly conserved in gregarine transcriptomes but present in both *P.* cf. *gigantea*.

#### Apicomplexan-specific glideosome proteins

The core glideosome machinery mainly comprises specialized proteins found only in apicomplexans. The single-headed short heavy chain myosin class XIV, named myosin A (MyoA), acts as a motor generating the rearward traction required for gliding motility, invasion and egress, as evidenced by various conditional depletion experiments [56–58]. The glideosome itself is situated between the plasma membrane and the apicomplexan-specific inner membrane complex (IMC). In the IMC, MyoA is associated with a light chain, myosin light chain 1 (MLC1) in *T. gondii* or MyoA tail domain-interacting protein (MTIP) in *P. falciparum* [59], as well as several glideosome associated proteins (GAP), GAP40, GAP45, GAP50 [60–62], GAP70 and GAP80 as yet only described in *T. gondii* [57]. GAP45 is thought to anchor the glideosome to the plasma membrane by recruiting MyoA as a bridge [62], whereas GAP40 and GAP50 are predicted to help anchor MyoA to the parasite cytoskeleton [63]. Another set of glideosome-associated proteins with multiple-membrane spans (GAPM) are believed to interact with the alveolin and subpellicular microtubules network, suggesting an indirect interaction with the IMC [26, 64]. Finally, the conoid-associated myosin H is necessary for initiating gliding motility in *T. gondii* [65].

Genes encoding myosins A, B, C, D and E and associated light chains were found in all species. Myosin H is also widely conserved in intracellular apicomplexans. However, among the gregarines Myosin H was only found in a few species. For glideosome associated proteins, only GAP40 was found in all species, although the sequences from gregarine transcripts and chromerids were less well conserved. Surprisingly, given the central role attributed to GAP45 in the glideosome model, no ortholog was found in gregarines except for two poorly conserved sequences in *Lankesteria abotti*, *Lecudina tuzetae*, *Cryptosporidium* and chromerids. However, we identified a short conserved 3′ domain (<50aa) in *L. tuzetae*, *Pterospora schizosoma* and *Siedleckia nematoides*. A similar domain is found in *P.* cf. *gigantea* A and B. It is however not sufficient to conclude whether it is an orthologous protein. GAP50 seems to be more conserved among apicomplexans, but is absent or only partially conserved in most of the gregarines. As expected, GAP70 and GAP80, only identified so far in *T. gondii*, were not found in other species, except for an orthologue of GAP80 in the coccidia *Hammondia hammondi*. Concerning GAPMs, we found orthologues of at least one of its variants (GAPM 1, 2 or 3) in most species. However, GAPMs seem to be totally absent in at least 7 species of gregarines (*Ancora sagittata, Protomagalhaensia* sp. Gyna*, Protomagalhaensia wolfi, Gregarina* sp. Pseudo*, Pterospora schizosoma, Selenidium pygospionis, Siedleckia nematoides*). Finally, GAC is overall well conserved in apicomplexans but absent from chromerids, supporting its apicomplexan-specific status. However, we were not able to identify GAC in several gregarine transcriptomes (*P.* sp. Gyna*, P. wolfi, G.* sp. Pseudo*, H. caprellae, L. abotti, L. tuzetae, P. schizosoma*) (Fig. 4A).

#### Adhesins and TRAP-like candidates

The glideosome machinery, anchored in the parasite cytoskeleton, needs to interact with extracellular receptors of the host cell to propel the parasite forward over the host surface. This is made possible by the presence of extracellular adhesins secreted by the micronemes [66, 67] and connected to the glideosome through the glideosome associated connector (GAC) protein [68]. Thrombospondin adhesive protein (TRAP) [69] is a *Plasmodium* adhesin required for gliding, whose homologue in *T. gondii* is MIC2 [70]. At the end of the gliding process, rhomboid protease 4 (ROM4) cleaves the adhesins, disengaging them from receptors and, for intracellular parasites, allowing them to enter the host cell [71–73]. TRAP-like proteins, while highly divergent from one species to another, constitute a family of functionally homologous proteins sharing adhesive domain types, involved in parasite motility and cell penetration [74–76]. TRAP-like or TRAP-related proteins have been detected in various stages of *Plasmodium* (CTRP [77], MTRAP [78], TLP [79]) and have also been found in silico in *Cryptosporidium* (TRAPCs, CpTSPs [76, 80, 81]) as well as in several *Babesia* and *Theileria* species [82–85], in *Neospora caninum* [86] and in *Eimeria* [87, 88]. We first looked for the TRAP proteins which have been implicated in gliding through experimental studies (MIC2, TRAP, TPL, CTRP, MTRAP), as well as the ROM4 protein involved in adhesin cleavage. Unsurprisingly, the currently described TRAP proteins seem to be genus- or even species-specific. On the other hand, we found orthologues for ROM4 in all species, except for chromerids.

The TRAP proteins described to date all have an extracellular region containing one or more TSP1 domains and/or one or more vWA domains [74–76]. They are also characterized by the presence of a single transmembrane domain, a signal peptide, and, in some cases, a juxtaposed rhomboid protease cleavage site, and a short, charged C-terminal cytoplasmic domain with aromatic residues. The presence of a YXXΦ tyrosine sorting signature has also been described [75] (where X signifies any amino acid, and Φ any hydrophobic amino acid).

**Fig. 5:**
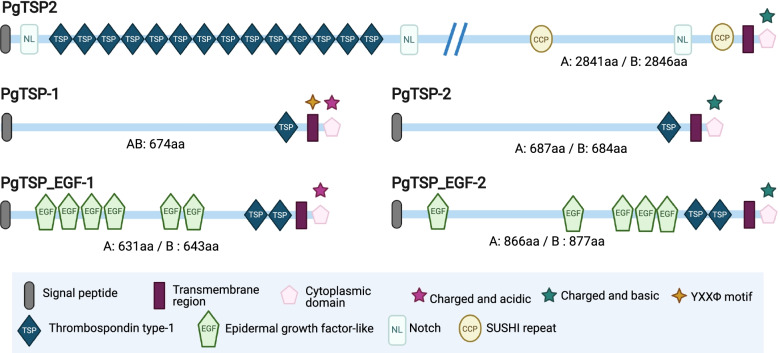
Structures and molecular domains of candidate TRAP-like proteins in *P.* cf. *gigantea* A and B. See also Table S8

To evaluate the presence of TRAP-like proteins in *P.* cf. *gigantea* genomes, we inventoried all predicted proteins containing at least one TSP1 domain (Table S8), then identified potential candidates with several TRAP-like structural characteristics (Fig. 5). We identified a CpTSP2 orthologue within both *P.* cf. *gigantea* genomes, designated PgTSP2. Like CpTSP2, it is a large protein (~ 2800 aa) composed of Notch, TSP1, and Sushi domains. PgTSP2 has a localization signal, a transmembrane domain and a short, charged, basic cytoplasmic tail. This protein also has orthologues in *G. niphandrodes,* in chromerids and coccidia.

We demonstrated the presence of genes encoding four other related protein pairs in both A and B genomes, most of which appear to be specific to *P.* cf. *gigantea.* PgTSP-1 has a TSP1 domain, a signal peptide, a transmembrane domain and a short, charged, acidic cytoplasmic tail. PgTSP-2, very similar in structure to PgTSP-1 also has a TSP1 domain, a signal peptide, a transmembrane domain, and a short, charged but basic cytoplasmic tail. PgTSP_EGF-1 has two TSP1 domains, a signal peptide, a transmembrane domain and a short, charged, acidic cytoplasmic tail, plus several extracellular EGF or EGF-like domains, as also described in *C. parvum* CpTSP7, CpTSP8 and CpTSP9 [80]. We identified another protein, PgTSP_EGF-2, very similar in structure.

#### Moving-junction associated proteins

In apicomplexans with intracellular stages such as *T. gondii*, invasion occurs when the extracellular tachyzoite initiates a pivotal movement known as reorientation, and a mobile junction settles into the host cell membrane allowing the parasite to enter. Gliding forces are also involved in this process [89], to which the host cell contributes [90]. A micronemal protein, AMA1, combines with rhoptry neck proteins (RON2, RON4, RON5 and RON8) to firmly secure the parasite to the host cell. In *P. falciparum,* another AMA-like protein, merozoite apical erythrocyte-binding ligand (MAEBL) has an important role in invasion alongside AMA1 [91].

Gregarines remain extracellular during their entire life cycle and *Cryptosporodium* display an intracellular but extra-cytoplasmic stage, so it was not surprising that we did not identify any orthologue of the moving-junction proteins of either these groups. We also searched for predicted proteins implicated in adherence and invasion in *Cryptosporidium*, such as GP15/40, GP900 and mucins, but found no equivalent in either *P.* cf. *gigantea* [92, 93].

#### Regulatory factors and signaling pathways

Increases in parasite intracellular calcium activate calcium-dependent protein kinases (CDPK) that regulate motility, microneme secretion, invasion and egress [94, 95]. Other proteins acting in such signaling pathways include diacylglycerol kinase 1 (DGK1) and acylated pleckstrin homology domain-containing protein (APH), which are involved in microneme secretion regulation [96, 97]; the C2 domain-containing protein DOC2.1 which mediates apical microneme exocytosis [98]; and the apical lysine methyltransferase (AKMT), which is involved in gliding motility, invasion and egress in *T. gondii* [99]. We were unable to identify APH in most gregarines and chromerids, and DOC2.1 could not be identified in several transcriptomes. All other regulatory factors appeared to be largely conserved.

## Discussion

### Molecular data support the presence of two species

We report here clear lobster coinfection by two gregarines believed to be distinct that we have named *Porospora* cf. *gigantea * A and *Porospora* cf. *gigantea *B. At the molecular level, these two organisms have very similar genomes in terms of size, protein coding capacity, GC content and overall organization with 86% synteny conservation. The delineation of species now requires more precise integrative morpho-molecular approaches, combining extensive imaging (SEM, TEM) and single cell –omics, to find specific traits. Currently, the only molecular tool available for species discrimination in gregarines is the nucleotide sequence of the 18S SSU rDNA. At this molecular marker level, *P.* cf. *gigantea* A and *P.* cf. *gigantea *B differ by a single nucleotide, a non-significant divergence for discriminating species. The main conclusion from this finding is that caution should be exercised when using 18S rDNA marker. The use of 18S rDNA marker is important and useful for placing a species in a phylogenetic tree, but it can also hide cryptic species, especially in eukaryotic microorganisms [100]. These cryptic species can also potentially have distinct adaptive abilities.

However, at the genomic level, the genomes show a nucleotide divergence of more than 10% which is incompatible with subspecies or strain definitions. By comparison, applying the same protocol to *P. falciparum* and *P. reichenowi* genomes concluded that the divergence between the two *Plasmodium* species is only 3.2%. Similarly, a divergence of 3–5% between the genomes of *C. parvum* and *Cryptosporidium hominis* has been reported [101]. The large overall genomic divergence between *P.* cf. *gigantea* A and *P.* cf. *gigantea* B indicates that they are probably not interfertile, and thus should be considered as different species.

Concerning their differential presence in lobster hosts, we observed the “lobster tank” specimen (*n* = 1) harbor only one parasite genome (i.e. *P.* cf. *gigantea* A) whereas “lobster bay” specimens (*n* = 2) were coinfected. Although we do have collected, over this sampling campaign, additional images for lobsters infected by *Porospora*, we can only speculate about the parasite species they harbor. Indeed, two parameters differentiate these two “lobster” sources. “Lobster bay” specimens are coming from Roscoff bay and have been raised in the wild whereas “lobster tank” specimen may in fact be coming from southern England and have later been maintained in captivity. Thereby, we have two strikingly different hypotheses to tentatively explain this observed difference in parasitic distribution. Firstly, we can propose that either both *P.* cf. *gigantea* A and *P.* cf. *gigantea* B are found in the wild (and both in Brittany and southern England) but only *P.* cf. *gigantea* A would survive a long captivity of its lobster host. Secondly, there could be an uneven distribution of *P.* cf. *gigantea* A and *P.* cf. *gigantea* B in the wild, depending on geography. Both species would co-occur near Roscoff while only or at least mostly *P.* cf. *gigantea* A, would occur in the wild in southern England. Regarding the simultaneous presence of two non-interfertile species within the same host, one hypothesis is that they could indeed inhabit different segments of the gut. Unfortunately, since the two species were identified after biological sampling, the resampling of several lobsters would be required. With the knowledge of the presence of two organisms, differences could be measured at the molecular level (by PCR, FISH or proteomics). Pending a more integrated morpho-molecular definition of their taxonomy, and better documentation of Cephaloidophoroidea species in general (Fig. 3), we propose that *P.* cf. *gigantea* A and *P.* cf. *gigantea* B are two distinct organisms infecting *H. gammarus*.

### Two species with compact genomes and a highly specific gene set in common

These two genomes are the first marine gregarine genomes to be sequenced and analyzed and the information generated considerably expands our knowledge of apicomplexan diversity. Both A and B genomes are very small compared to other apicomplexans, with a particularly high gene density. For example, for a similar genome size, *Cryptosporidium* spp. have only about 3900 protein-coding genes compared to the 5300 genes of *P.* cf. *gigantea*. This result could be partially explained by the absence of certain non-coding sequences in the assemblies, such as centromeres, telomeres and repeated sequences which are difficult to sequence and assemble, notably in de novo assembled genomes. However, the compaction is partially due to the comparatively short introns. Small introns with similar consensus sequences have been described in *Babesia microti* [31].

So far, we have not found any evidence of organellar genomes, whether from the mitochondrion or apicoplast. This needs to be investigated more definitively, especially the mitochondrial aspects. Indeed, the cystic stages from which DNA was collected are unlikely to have many mitochondrial genome copies. To address this issue, it would be more suitable to investigate trophozoite stages via single-cell genomics, for example. According to a recent study, mitochondrial genomes seem to have disappeared from eugregarines [32]. Instead of a distinct mitochondrial genome, the 129 mitochondrial proteins differentially conserved among the gregarine lineages are encoded in the nuclear genome. It would be interesting to identify how many of these nuclear-encoded proteins are conserved within the *P.* cf. *gigantea* genomes and to reconstruct their specific metabolism. Regarding the apicoplast genome, a recent study stated that it has probably been lost in all eugregarines, while archigregarines may have conserved a highly reduced plastid genome [10].

BUSCO genome completeness scores of ~ 70% were found for the two *P.* cf. *gigantea* genomes, a value not unusual for non-model species [29], but lower than was found for the *G. niphandrodes* genome (83%) and the 24 other representative species we evaluated (from 76.9% for *Cystoisospora suis* to 100% for *P. falciparum* (Fig. S4)). This result also illustrates that the definition of “Apicomplexa core genome” is probably currently highly biased, notably towards *Plasmodium*. Gregarines should be taken into more consideration, as their divergence compared to other apicomplexan models was confirmed by the orthogroup analysis indicating a low percentage of genes conserved between A or B and other studied apicomplexans (< 18%).

Even among gregarines the wide diversity is noted as the vast majority of proteins shared by A and B are absent from the *G. niphandrodes* genome. Therefore, studying gregarines will allow a better understanding of the evolutionary history of apicomplexan species, and highlight the astonishing protein diversity brought about by complex differential inheritance from the common ancestor. Through comparative analyses, we will be able to understand how this inheritance has allowed such a wide range of adaptations to parasitism in apicomplexans, which have been able to establish themselves in most Metazoan lineages, vertebrate or invertebrate, marine or terrestrial, in one or more hosts, intracellular or extracellular modes.

### The gregarine glideosome(s)

#### An incomplete but operational machinery

Gliding motility is an essential feature of apicomplexans, and for some intracellular parasites among them, glideosome proteins have been shown to be crucial for host cell invasion and egress [22, 23, 26, 63, 74]. However, our sequence analysis of the glideosome components shows that the currently known mechanistic model based on *T. gondii* and *P. falciparum* does not fully account for gliding in all apicomplexans, as anticipated [26, 63, 67]. Moreover, the conservation of the proteins involved is very variable among the gregarines for which we have omic data. Although it is possible that those proteins may be so divergent from a species to another that current data mining methods are unable to retrieve them, the little evidence we were able to find of key molecular components such as canonical adhesins or GAP45 suggests that gregarines and *Cryptosporidium* species may have an at least partially alternative machinery dedicated to gliding (Fig. 4B), especially since *P.* cf. *gigantea* trophozoites are able to glide so rapidly.

#### The model machinery may be partially compensated by alternative proteins

The TRAP adhesin in *T. gondii,* named TgMIC2, has been demonstrated to be an important but non-essential protein to motility [102]. This suggests that TRAP proteins may not be the only proteins involved in host surface adhesion. As we have seen, in the genomes of *P.* cf. *gigantea* and in other apicomplexans, there are proteins with a structure similar to TRAPs (TRAP-like), that might replace the canonical TRAPs. Understanding the evolution of TRAP requires experimental validation of predicted adhesion proteins in gregarines and *Cryptosporidium* - especially since the presence of these domains in Alveolata does not always correlate with gliding motility [76]. Similarly, the vWA domains, which are found in the canonical TRAPs, appear to be absent from the *Cryptosporidium* genomes. Since gliding is observed in *Cryptosporidium* species, it can be assumed that, if the TRAP-like proteins described in *Cryptosporidium* are indeed involved in gliding, then the vWA domains are not essential for this process. It is also possible that the TSP1 domain proteins represent only one adhesion pathway among others, and that other adhesion domains could perform functions similar to TRAPs, such as the Apple and EGF-like domains in *Cryptosporidium* [75, 80]. This is a plausible idea since ROM4, which cleaves adhesins from extracellular receptors of the host cell at the end of the gliding process, is extremely well conserved. GAP45 is thought to maintain the interaction between the IMC and the plasma membrane, and acts as an essential bridge between the two structures [103]. Deleting GAP45 has been proved to prevent glideosome assembly in *P. falciparum* [104]. Perhaps the absence of GAP45 in gregarines and *Cryptosporidium* could be compensated by other GAP-like proteins or it may not even be necessary. Indeed, a looser motor architecture has been proposed, in which actin-myosin motors push in a general backward direction, without necessarily being guided by GAP proteins [63]. Furthermore, while TgMLC1 binding to TgGAP45 is considered a key component of the parasite’s force transduction mechanism, it has recently been shown that loss of TgMLC1 binding to TgGAP45 has little effect on their ability to initiate or maintain movement [105], questioning again the real role of GAP45 and suggesting our comprehension of the intricacies of the glideosome is still incomplete.

#### Different structures for other forms of gregarine motility?

Gregarines have other means of motility, presumably governed by other molecular mechanisms, and the relevance of the glideosome concept to gregarines has been questioned [27, 106]. In particular, archigregarines use several modes of movement such as rolling and bending, but not gliding [6, 19]. Coelomic and intestinal eugregarines, like crustacean gregarines, have longitudinal, drapery-like surface structures called epicytic folds, the most distinctive feature that differentiates eugregarine trophozoites and gamonts from other apicomplexans. These structures are considered to be involved in eugregarine gliding by increasing the surface area and facilitating actomyosin-based gliding motility, reviewed in Valigurová et al. (2013) [27]. Indeed, actin and myosins A, B and F have been localized in epicytic folds in *Gregarina polymorpha* [107, 108]*.* Epicytic folds and mucus, the substance often observed in the trace left by gliding eugregarines [6, 27], are key components to integrate into an alternative model to the current glideosome more representative of eugregarine motility. A particularly interesting study of the crustacean gregarine *Cephaloidophora* cf. *communis* reported specific attachment apparatus structures [109]. While actin in its polymerized form (F-actin) is observed all along the gregarine, myosin is confined to the cortical region of the cell, in connection with the longitudinal epicytic folds, as described in Valigurová et al. (2013) [27]. This organism also has also a septum, a tubulin-rich filamentous structure that separates the epimerite from the protomerite at the cell apex. Together with microneme-like structures, these features suggest adhesion proteins are produced which could be threaded through the membrane by the numerous pores visible on the epimerite [109]. We were unable to identify alternative movements to gliding in *P.* cf. *gigantea* (such as peristaltic movement described in other coelomic eugregarines [6, 110]). Additional observations are needed to fully document the range of potential motilities in this species, especially since the crustacean-infecting gregarine *C.* cf. *communis* is capable of jumping or jerking during discontinuous gliding [109]. The different structures invoked, or their absence must be evidenced; indeed, in eugregarines, subpellicular microtubules have never been observed, even though they are supposed to be involved in gliding motility in other apicomplexans [27, 109]. An important point to remember is that eugregarines and archigregarines remain mostly extracellular and do not invade the parasitized cell, unlike intracellular parasites like *T. gondii* or *P. falciparum*. Moreover, archigregarines have very well-structured apical complexes containing most of the elements described in intracellular Apicomplexa (rhoptries and micronemes) but are not endowed with gliding capacities. On the other hand, eugregarines such as *Porospora* are capable of gliding motility but usually have much smaller apical complexes [6]. In *Porospora* zoites, it is reduced to a small conical structure, and dense inclusions in the cytoplasm anterior to the nucleus that may correspond to rhoptries and micronemes [18]. The precise role of the apical complex and gliding motility in eugregarines needs further studies, but whatever will be the mechanisms sustainging such abilities in *P.* cf. *gigantea,* there are likely to be unique molecular structures, which have evolved consecutive to the specific evolutionary path of gregarines, and which differ from what is currently documented in other apicomplexan lineages [26]. We hope that this work will pave the way for in silico and experimental studies to further characterize the specific protein pool of gregarines and to reveal their functional potential for a better understanding of important apicomplexan abilities such as gliding motility.

## Conclusion

In this work, we have characterized for the first time not one but two genomes for a marine eugregarine, thus providing a major contribution regarding the coding capacities of these early branching apicomplexans, and a much-needed insight for better understanding apicomplexan adaptive capacities and evolutionary history. These two marine gregarine genomes are reduced, as are most apicomplexan genomes, but still have retained a significant number of genes, suggesting they have important and diversified requirements to fulfill their complete life cycle.

A major outcome of the whole genome comparative studies was the discovery that these marine eugregarine genomes were as distantly related to the only other genome known for a gregarine, that of *Gregarina niphandrodes*, as they are to any other apicomplexan genome. This indicates, first, that it will be essential to study many additional gregarine genomes before having an exhaustive, accurate view of the true diversity of these early diverging apicomplexan groups. Second, it reveals that the exploration of these future gregarine genomes promises to uncover an astonishing number or novel, currently totally unknown protein diversity for the apicomplexan phylum, as *Porospora* and *G. niphandrodes* share only a fifth of their orthologs. We do hope that this pioneering work on *Porospora* will help pave the way for this much needed exploration of the coding capacities of a much wider diversity of early diverging members of the phylum Apicomplexa.

Our exploration of the proteins involved in gliding motility, an emblematic feature of apicomplexans, has started to illustrate some outcomes provided by comparative genomics across a wider diversity of Apicomplexa. We found a high conservation of actin-related proteins and regulatory factors within apicomplexans, in sharp contrast to a highly variable conservation of some central glideosome proteins and of all adhesins across apicomplexan lineages.

Our work illustrates the importance of studying gregarines to broaden our biological and evolutionary view of apicomplexan parasites, to better understand the breath of diversity they cover, and to extend our understanding of the molecular basis of some key apicomplexans features, such as the gliding motility.

## Material & methods

### Experimental model and subject details

Several specimens (*n* = 35) of the lobster species *Homarus gammarus* were collected in the English Channel at Roscoff (Brittany, France) between July 2015 and October 2017 (Table S2), either directly from the wild (Roscoff Bay) or from lobster tank facilities, in which crustaceans are maintained in captivity several weeks to months before their commercialization. According to UICN Red list, *Homarus gammarus* is not an endangered species [111]. The intestinal tract was carefully dissected from each freshly killed host specimen, and transferred to large Petri dishes filled with 0.22-μm filtered, autoclaved sea water, supplemented with the antibiotics penicillin (100 U/mL), streptomycin (100 μg/mL) (Gibco, Life Technologies, USA) and gentamycin (50 μg/mL) (Interchim, Montluçon, France). Trophozoites freely moving in the upper intestine lumen, and cysts loosely attached within the chitinous folds of the hosts’ rectal ampullae (Fig. S1), were individually collected using elongated Pasteur pipettes under a classic binocular microscope. For the recording of gliding movement, trophozoites were kept in non-treated sea water. For all other methods, trophozoites, cysts and host tissues were carefully washed several times in 0.22-μm filtered, autoclaved sea water supplemented with the antibiotics indicated above. Trophozoites and cysts were collected for photonic live imaging, scanning electronic microscopy and transmission electronic microscopy, as well as for subsequent omics studies (i.e. DNA and RNA sequencing).

## Method details

### Electronic microscopy

For the scanning electron microscopy (SEM) studies, isolated trophozoites and cysts, or host intestines and rectal ampullas opened along their longitudinal axis, were washed as indicated above then fixed in 2.5% (v/v) glutaraldehyde in 0.1 M sodium cacodylate (pH 7.2) at 4 °C for 6 to 12 hours. After two washing steps in 0.1 M sodium cacodylate (pH 7.2), biological specimens were transferred to microporous specimen capsules (30 μm porosity, 12 mm diameter, 11 mm high, ref. #70187–20, Electron Microscopy Science) and dehydrated in a graded series of ethanol in double-distilled water (50, 70, 90, and 100%). Biological specimens in the capsules were critical point-dried in liquid CO_2_ (Emitech K850, Quorum Technologies), then transferred to adhesive carbon-coated holders, and coated with 20 nm of gold (JEOL Fine Coater JFC-1200). Specimens were then examined with a Hitachi SU3500 Premium scanning electron microscope.

For the transmission electron microscopy (TEM) studies, samples were fixed for 2 h in 0.2 M sodium cacodylate buffer with 4% glutaraldehyde, 0.25 M sucrose in 0.2 M sodium cacodylate buffer pH 7.4. Cells were then washed three times in sodium cacodylate buffer containing decreasing concentrations of sucrose (0.25 M, 0.12 M, 0 M) for 15 min each time, followed by post-fixation for 1 h at 4 °C in 2% osmium tetroxide in 0.1 M sodium cacodylate buffer. After three rinses in 0.2 M sodium cacodylate buffer, samples were dehydrated by successive transfer through an increasing ethanol series (25, 50, 70, 90%, 3 × 100%), then embedded in Spurr’s resin. Sections were cut using a diamond knife on a Leica Ultracut UCT ultramicrotome (Leica, Wetzlar, Germany) and after staining with saturated uranyl acetate for 15 min and Reynolds’ lead citrate for 3 min, were examined on grids with a Jeol 1400 transmission electron microscope (Jeol, Tokyo, Japan).

### DNA/RNA isolations

Genomic DNA (gDNA) was isolated from 4 biological samples of pooled cysts taken from 3 specimens of the host *H. gammarus*: sample JS-470 from Lobster #7 (~ 70 cysts), sample JS-482 from Lobster #11 (~ 50 cysts), samples JS-488 and JS-489 from Lobster #12 (~ 100 cysts each). Lobster #7 was provided by the Roscoff lobster tank facility while Lobster #11 and Lobster #12 were caught from Roscoff bay. DNA was extracted from the pooled cysts using Macherey Nagel Tissue and Cells isolation kit (ref 740,952.50) with yields of 4.1 μg (JS-470), 2 μg (JS-482), 4.5 μg (JS-488) and 6.7 μg (JS-489) of total DNA per sample, as measured by Nanodrop quantification. The protocol was used as recommended by Macherey Nagel, except that the initial lysis step at 56 °C was extended beyond the recommended to 1–3 hours with frequent microscopic (binocular) inspection to monitor cyst digestion until completion.

RNA was also isolated from 2 additional biological samples, both composed of pooled cysts taken from the rectal ampulla of their respective hosts: JS-555 (~ 35 cysts, Lobster #26, Roscoff bay) and JS-575c (~ 40 cysts, Lobster #34, Roscoff Lobster tank facility). Two distinct protocols were used to isolate total RNA from these two biological samples. For sample JS-555, we used Macherey Nagel basic RNA Isolation kit (ref 740,955.10) which yielded ~ 155 ng of total RNA in 55 μl as assessed by Qbit quantification. For sample JS-575c, we used Macherey Nagel Nucleozol-based RNA Isolation kit (refs 74,040.200 and 740,406.10) which yielded ~ 50 ng of total RNA in 55 μl as assessed by Qbit quantification.

### DNA/RNA sequencing and assembly

The gDNA extracted from the 4 biological samples (JS-470, JS-482, JS-488 and JS-489) was sequenced individually using Illumina NextSeq technology (2 × 151 bp; NextSeq 500 Mid Output Kit v2; Institut du Cerveau et de la Moelle - CHU Pitié-Salpêtrière - Paris). We obtained 2 × 50 M to 2 × 70 M reads which were checked using FastQC [112] (version 0.11.5). Reads were cleaned with Trim Galore [113] (version 0.4.4) which removed remnant Nextera adaptors, clipped 15 bp at 5′-ends and 1 bp at 3′-ends and trimmed low-quality ends (phred score < 30). The assembly was carried out using SPAdes [114] (version 3.9.1; options: careful mode, automatic k-mers) with the pooled libraries (Fig. S2.A).

RNA was extracted from both samples (JS-555 and JS-575c) and treated with RNAse-free DNase. Libraries (Institut du Cerveau et de la Moelle - CHU Pitié Salpétrière - Paris) were prepared following the kit manufacturer’s recommendations (SMART-Seq v4 Ultra Low Input RNA Kit from Takara). Samples were sequenced on a NextSeq 500 Illumina device with MidOutPut cartridge to generate a total of 2 × 87 M reads of 75 bp. The read quality was checked by using FastQC and cleaned by using Trim Galore to remove remnant Nextera adaptors, clipping 15 bp at 5′-ends and 1 bp at 3′-end and trimming low-quality ends (phred score < 30). The sequence reads of both samples were merged into one library which was assembled using Trinity [115, 116].

All genomic contigs longer than 1 kb were analyzed by principal component analysis (PCA) based on their 5-mer composition, which classified them into 6 groups using a hierarchical clustering method (HCA) based on the Ward criterion (Fig. S2.B).

For all contigs, the putative protein coding genes were then predicted using Augustus [117] (version 3.3) and the Apicomplexa gene model for *T. gondii*. All the predicted proteins were thus compared with the NCBI non-redundant protein database using BLAST [118]. The analysis of the taxonomic groups corresponding to the best hits, enabled us to identify five clusters as putative bacterial contaminants whereas the sixth cluster which included 1745 contigs (18.0 Mb), was identified as organisms closely related to Apicomplexa, referred to as the “apicomplexa” cluster (Fig. S2.B).

### Identification of genomes A and B

Preliminary analysis of the “apicomplexa” cluster exhibit two sets of contigs with approximatively 10% of divergence and specific coverage values in the four libraries. The contigs of the “apicomplexa” cluster were split into genomes A and B by using the difference in coverage observed for the four gDNA libraries (Fig. S2.C, Fig. S3). Each gDNA library (JS-470, JS-482, JS-488 and JS-489) was individually mapped to the contigs using Bowtie2 [119] and the median coverage was calculated for each contig and each library using Samtools [120] and Bedtools [121] suites. This coverage information was processed by PCA and a k-means algorithm which classified the contigs into 2 clusters. Then, a linear discriminant model was trained with the coverage information and the result of this first classification before applying it to all the contigs in order to improve the classification. The linear discriminant method (training and classification) was iterated 3 times until convergence. A similar analysis was carried out with 1-kb non-overlapping windows (instead of full-length contigs) to identify putative hybrid contigs. Contigs were thus classified to different genomes depending on the windows, then divided into sub-contigs which were re-assigned to their respective genomes. A detailed protocol with R scripts is available on github (see data and code availability).

The nucleotidic divergence between genome A and genome B was estimated from the alignment of contigs built with Mummer3.0 [122]. All alignments of the syntenic regions were parsed to compute the divergence using a home-made script. Assembly metrics were assessed by using QUAST [123] (version 5.0).

### Prediction and annotation

All de novo assembled transcripts were aligned against the “apicomplexa” cluster contigs with GMAP [124] within the PASA program [125]. Then, two ab initio gene prediction tools, SNAP [126] (version 2017-11-15) and Augustus were trained using a subset of the PASA transcriptome assemblies. A specific gene model was trained with Augustus, including meta-parameter optimization and prediction of introns (allowing small intron length > 10 bp) using our “apicomplexan” cluster repeat-masked genome assembly as reference (RepeatMasker [127], version 4.0.8). Gene predictions were then performed allowing for the prediction of alternative transcripts and noncanonical intron splice sites. An alternative model was also trained with SNAP (default protocol) and used for gene predictions. The Augustus and SNAP outputs showed that some gene predictions were slightly different, so the predictions were parsed with a home-made script to keep as many alternative genes and transcripts as possible for each prediction made. The completeness of the gene prediction was assessed using BUSCO (version 4.0.6).

The predicted proteins were automatically annotated by using i) the best hit of a BLASTP search against VEupathdb (version 2019-01-20), ii) the results of KoFamScam against the KEGG pathway database [128] (version 2019-05-11) and iii) the signature domains obtained with Interproscan [129] (version 5.39–77.0).

The ortholog groups were identified with orthoMCL [130] (default parameters, version 2.0.9) applied to the proteome of a selection of representative organisms available on VEuPathDB (Table S1).

The divergence time of genome A and genome B was estimated from the divergence time of *P. falciparum* and *P. reichenowi* as estimated in TimeTree. Then the coding sequences of the orthologous groups/quartets including a single gene each for genome A, genome B, *P. falciparum* and *P. reichenowi* were aligned using MacSE [131]. For each alignment, the number of synonymous substitutions per site (dS) between genomes A/B and between *P. falciparum/reichenowi* were computed with the maximum likelihood method of Yang and Nielsen (2000) [132] implemented in PAML4 [133].

The Infernal software [134] (version 1.3.3) and the Rfam database [135] (version 14.2) were used together to search for transfer RNAs, spliceosomal RNAs and ribosomal RNAs. The snoReport software [136] (version 2) was used to search C/D and H/ACA small nucleolar RNAs.

### Removal of contaminant sequences

#### Host contaminants

All “apicomplexa” cluster contigs were screened against the short reads available from the *Homarus americanus* (PRJNA486050) genome sequencing project, to identify closely-related host contaminants. This dataset was assumed to be free of sequences from apicomplexans, since it was obtained from DNA extracted from non-intestinal tissues (tail, leg or pleiopod appendices). Mapping was carried out with Bowtie2 and the coverages were calculated by using Samtools. The contigs thus identified that were covered over more than 60% of their length by *Homarus* short reads, were considered as host contaminants and were removed.

#### Prokaryotic and fungal contaminants

In parallel, predicted genes in the “apicomplexa” cluster contigs were deeply analyzed for the presence of bacterial and fungal sequences. For each scaffold containing at least one predicted protein, a BLASTP against the NCBI NR database was launched. If the resulting hit had an e-value lower than 1e-30 and more than 30% of the length of the contig was covered by prokaryote/fungi hits, an additional BLASTN against NCBI NR/NT was performed. For the remaining scaffolds without predicted proteins, a direct BLASTN vs NR/NT search was performed. At the end of this procedure, the contigs with prokaryotes/fungi hits covering more than 70% of the length were labeled as contaminants and were removed from the genome assembly.

### Search for organellar genomes

Organellar genomes were searched using the mitochondrial or apicoplastic genomes available in VEupathDB (version 2019-01-20) as well as with the contigs described in Janouškovec et al. (2019) [10] as reference sequences. Firstly, a similarity search using a TBLASTX and these sequences as query was applied on all assembled contigs (identified as *P. gigantea* or not). All hits with a bit score above 100 were considered as organellar candidates and were extracted (with 100 bp upstream and downstream). Secondly, these candidates were used in a reciprocal TBLASTX search against NCBI NR database to eliminate bacterial contamination. The regions exhibiting at least one hit against an eukaryotic sequence among the nine best hits were manually studied to check if the associated contigs could correspond to organellar genomes.

### Experimental reconstruction of 18S/28S loci

First, a partial SSU rDNA locus was amplified by using JS-470 gDNA (i.e. genome A only) as template and WL1 and EukP3 primers (Table S7) in a conventional PCR reaction. The amplified bands were cloned and sequenced as previously described [40]. The resulting partial SSU rDNA sequence was further extended in the 3′ direction still using JS-470 gDNA as template and novel primers designed or re-designed based on the molecular data published for *Cephaloidophora* cf. *communis* and *Heliospora* cf. *longissima* [39] (Fig. S6.A). The resulting sequence (> 4 kb) was then used as anchor to reconstruct a complete ribosomal locus with the program iSeGWalker [137]. By clustering reads from JS-470 on this anchor, a 7322-bp theoretical sequence that corresponded to [partial 28S – 18S – ITS1–5.8S – ITS2 – partial 28S] including a perfect 1352-bp overlap between the 5′ and 3′ [partial 28S] segments was obtained. From this a complete ribosomal locus [18S – ITS1–5.8S – ITS2 –28S] of 5977 bp for genome A was reconstructed, which was validated by PCR amplification, cloning and sequencing (Fig. S6.B). In a similar clustering approach using all reads for JS-482, JS-488 and JS-489, the complete ribosomal locus for genome B was reconstructed in silico, which is the same length but has 30 polymorphisms compared to the genome A locus (Fig. S6.C). Next, 50% of the complete ribosomal locus for genome B was confirmed by PCR amplification, cloning and sequencing (positions 1187 to 4217, covering partial 18S-ITS1–5,8S-ITS2-partial 28S). This second round of clustering was also used to quantify the respective distributions of genomes A and B present in the latter three biological samples at the full ribosomal locus level. The validated sequence of 18S/28S was manually added to the genome assemblies of genomes A and B, respectively. Schematic representation of rRNA loci was done using BioRender (biorender.com).

### Phylogeny

#### Phylogenomics of gregarines

The phylogenomic tree was built from a super matrix of 312 orthologues from two datasets published by Salomaki et al. (2021) [13]. These two datasets are composed by 246 and 299 orthologues respectively. For all orthologues, corresponding genes have been searched in the proteomes of *P.* cf. *gigantea* A and B by using BLASTP and candidates were aligned with known orthologues using mafft [138]. Then, orthologous relationships were validated by visual inspection of all the single-protein phylogenetic tree using RaxML [139] with rapid bootstraps (−f a), −m PROTGAMMAAUTO. Orthologues for *P.* cf. *gigantea* A and/or B have been recovered for 201 and 256 orthologues in both initial datasets. Both datasets were grouped into a larger dataset composed by 312 non-redundant orthologues. All orthologues were I) filtered with Prequal [140] to remove non-homologous residues, ii) aligned with mafft, iii) filtered with divvier [141] to remove alignment errors, iv) trimed with trimAl [142] and v) merged into the super matrix by using the script *matrix_constructor*.*py* available with PhyloFisher [143]. The maximum likelihood tree was built with IQ-Tree2 under LG + C60 + G + F [144]. The reliability of the phylogenetic tree was tested by the SH-aLRT and ultrafast bootstrap methods (repeated 1000 times). Bayesian phylogenetic tree was constructed with MrBayes [145] (version 3.2.3) using a LG + G + F model on a partitioned alignment: prset applyto = (all) aamodelpr = fixed(lg); prset applyto = (all) statefreqpr = fixed(empirical); lset applyto = (all) rates = gamma; unlink shape = (all) pinvar = (all) statefreq = (all); mcmc ngen = 500,000 samplefreq = 1000 printfreq = 10,000 nchains = 4 nruns = 2 savebrlens = yes; sump burnin = 25,000; sumt burnin = 25,000 contype = allcompat. All trees were visualized and edited using FigTree [146] (version 1.4.4) and Inkscape (www.inkscape.org).

#### 18S phylogeny of gregarines

The 100-sequence phylogeny was built from the 18S SSU rDNA sequences of the two genotypes of *P.* cf. *gigantea*, which were aligned with 84 sequences from a diversity of gregarines, either marine or terrestrial, as well as 12 other apicomplexan sequences. Two chromerid sequences were used as the outgroup [147] but several trees including more than 20 sequences selected from a large diversity of outgroups (from Cryptosporidians, Coccidians, Hematozoans, Colpodellids, Chromerids, Perkinsids, Dinoflagellates, Ciliates, Colponemids, Heterokonts and/or Rhizaria) were built based on Schrével et al. (2016) [40] and conducted to the same conclusions. A total of 1614 sites were found to be conserved after selecting conserved blocks as defined by Gblocks [148] (version 0.91b) with the following parameters: minimum number of sequences for a conserved position, 51; minimum number of sequences for a flanking position, 51; maximum number of contiguous non-conserved positions, 8; minimum length of a block, 3; allowed gap positions, all. A general time reversible (GTR) substitution model with gamma-distributed rate variation across sites and a proportion of invariant sites was suggested as the best-fit model according to the Bayesian information criterion (BIC) and the Akaike information criterion (AIC) calculated by MEGA X [149]. Maximum likelihood analyses were performed using RAxML (version 8.2.12) with bootstraps estimated from 1000 replicates. A Bayesian phylogenetic tree was constructed with MrBayes (version 3.2.3) using the following parameters: lset nst = 6 rates = invgamma; mcmc ngen = 10,000,000, relburnin = yes burninfrac = 0.25, samplefreq = 1000, printfreq = 10,000, nchains = 4, nruns = 2, savebrlens = yes; sump burnin = 2,500,000; sumt burnin = 2,500,000, contype = allcompat.

#### Environmental 18S phylogeny focused on crustacean gregarines

The 189-sequence phylogeny was built from the 18S SSU rDNA sequences from genomes A and B aligned with 14 from crustacean gregarines, and 154 environmental sequences from several projects described in Rueckert et al. (2011) [41] or gathered from NCBI Genbank. The sequences from the Gregarinoidae clade (*n* = 19) were used as the outgroup, as this clade has been placed as a sister group to the crustacean gregarine clade in recent literature [10–12]. A total of 1135 sites were found to be conserved after selecting conserved blocks as defined by Gblocks with the following parameters: minimum number of sequences for a conserved position, 95; minimum number of sequences for a flanking position, 95; maximum number of contiguous non-conserved positions, 8; minimum length of a block, 3; allowed gap positions, all. Maximum likelihood and Bayesian analyses were performed following the same protocol and parameters as in the previous 18S phylogeny.

### Expert annotation for glideosome proteins

A reference apicomplexan glideosome protein dataset was written based on glideosome protein repertoires described in the literature mainly for *T. gondii* and *P. falciparum* [26, 63, 67]. This reference dataset was used as a seed for parsing the orthogroups established for 25 reference proteomes (Table S1) and the predicted proteomes of the two *P.* cf. *gigantea* genomes. These reference proteomes were selected by considering the most recent data and associated publications to have the most complete panorama of apicomplexan proteins and key functions/structures documented to date. We also searched for potential orthologues within all recently published proteomes of gregarines using BLASTP (seed: reference proteins in *T. gondii* and *P. falciparum*).

For each orthogroup containing at least one of the reference proteins, the list of proteins was extracted, and the protein sequences were recovered with their respective coding sequences for both *P.* cf. *gigantea* genomes. BLASTP was performed for extracted proteins against the proteomes of *P.* cf. *gigantea*, as well as for the candidate proteins from each *P.* cf. *gigantea* genome against the 25 species reference proteomes. BLASTN was performed against NCBI NR for the coding sequences of the candidate proteins of both *P.* cf. *gigantea* genomes. The sequences thus collected for each described protein were aligned with mafft. Maximum likelihood molecular phylogeny was deduced from each alignment using RAxML. Analyses were performed using the LG model; bootstraps were estimated from 1000 replicates. Annotations of the conserved molecular domains were searched for in the automatic annotation and structure analyzed with SMART [150]. For each protein, the results of all the analyses were examined to validate the candidate proteins within the proteomes of the two *P.* cf. *gigantea* genomes. A table summarizing the presence or absence of glideosome proteins was visualized using R using the tidyverse package [151]. Putative TRAP-like proteins were identified by searching for sequences encoding the TSP1 molecular domain (IPR000884) within the two *P.* cf. *gigantea* genomes. The predicted structure of each candidate protein was studied, and if necessary partially predicted proteins were re-edited with Genewise [152]. Schematic representation of TRAP-like proteins was done using BioRender (biorender.com).

## Supplementary Information

**Additional file 1.**

**Additional file 2.**

## Data Availability

DNA and RNA reads and genome assemblies are available in the NCBI database [Bioproject PRJNA734792)]. Detailled protocols as well as complementary data (scanning electron microscope images, phylogenomics datasets, alignments, phylogenetic trees, blasts results and orthogroups) are available on Github [https://github.com/julieboisard/Marine_gregarines_genomes.git/].

## References

[1] Adl SM, Bass D, Lane CE, Lukeš J, Schoch CL, Smirnov A, Agatha S, Berney C, Brown MW, Burki F (2019). Revisions to the classification, nomenclature, and diversity of eukaryotes. J Eukaryot Microbiol.

[2] Swapna LS, Parkinson J (2017). Genomics of apicomplexan parasites. Crit Rev Biochem Mol Biol.

[3] Portman N, Šlapeta J (2014). The flagellar contribution to the apical complex: a new tool for the eukaryotic Swiss Army knife?. Trends Parasitol.

[4] Boisard J, Florent I (2020). Why the –omic future of Apicomplexa should include gregarines. Biol Cell.

[5] del Campo J, Pons MJ, Herranz M, Wakeman KC, del Valle J, Vermeij MJA, Leander BS, Keeling PJ (2019). Validation of a universal set of primers to study animal-associated microeukaryotic communities. Environ Microbiol.

[6] Desportes I, Schrével J, editors. Treatise on zoology--anatomy, taxonomy, biology: The Gregarines. The early branching Apicomplexa. Leiden: Brill; 2013.

[7] Rueckert S, Betts EL, Tsaousis AD (2019). The symbiotic Spectrum: where do the gregarines fit?. Trends Parasitol.

[8] Templeton TJ, Enomoto S, Chen W-J, Huang C-G, Lancto CA, Abrahamsen MS, et al. A genome-sequence survey for *Ascogregarina taiwanensis* supports evolutionary affiliation but metabolic diversity between a gregarine and *Cryptosporidium*. Mol Biol Evol. 2010;27:235–48. 10.1093/molbev/msp226 PMC2877549 19778951

[9] Aurrecoechea C, Barreto A, Basenko EY, Brestelli J, Brunk BP, Cade S, Crouch K, Doherty R, Falke D, Fischer S (2017). EuPathDB the eukaryotic pathogen genomics database resource. Nucleic Acids Res.

[10] Janouškovec J, Paskerova GG, Miroliubova TS, Mikhailov KV, Birley T, Aleoshin VV, Simdyanov TG (2019). Apicomplexan-like parasites are polyphyletic and widely but selectively dependent on cryptic plastid organelles. eLife.

[11] Mathur V, Kolísko M, Hehenberger E, Irwin NAT, Leander BS, Kristmundsson Á, Freeman MA, Keeling PJ (2019). Multiple independent origins of apicomplexan-like parasites. Curr Biol.

[12] Mathur V, Kwong WK, Husnik F, Irwin NAT, Kristmundsson Á, Gestal C, Freeman M, Keeling PJ (2021). Phylogenomics identifies a new major subgroup of apicomplexans, Marosporida *class nov.*, with extreme Apicoplast genome reduction. Genome Biol Evol.

[13] Salomaki ED, Terpis KX, Rueckert S, Kotyk M, Varadínová ZK, Čepička I, Lane CE, Kolisko M (2021). Gregarine single-cell transcriptomics reveals differential mitochondrial remodeling and adaptation in apicomplexans. BMC Biol.

[14] Beneden V. Sur une nouvelle espèce de Grégarine désignée sous le nom de *Gregarina gigantea*. Bulletins de l’Académie Royale de Belgique. 1869;28:444–56.

[15] Schneider A (1875). Contribution à l’ histoire des Grégarines des Invertébrés de Paris et de Roscoff. Arch Zool Exp Gen.

[16] De Bauchamp P. Sur une grégarine nouvelle du genre *Porospora*. C R Acad Sci Paris. 1910;151:997–9.

[17] Hatt P. L’évolution des Porosporides chez les mollusques. Archives de zoologie expérimentale et générale. 1931;72:341–415.

[18] Desportes II, Theodorides J. Ultrastructure of the Gymnospore of *Porospora* (Eugregarina, Porosporidae). C R Acad Sci Paris. 1965;260:1761–2. 14341296

[19] Schrével J, Desportes I. Gregarines. In: Mehlhorn H, editor. Encyclopedia of parasitology. Berlin Heidelberg: Springer; 2015. p. 1–47.

[20] Russell DG. Host cell invasion by Apicomplexa: an expression of the parasite’s contractile system? Parasitology. 1983;87:199–209. 10.1017/s0031182000052562 6646806

[21] King CA. Cell motility of sporozoan protozoa. Parasitol Today. 1988;4:315–9. 10.1016/0169-4758(88)90113-5 15463014

[22] Sibley LD, Håkansson S, Carruthers VB. Gliding motility: An efficient mechanism for cell penetration. Curr Biol. 1998;8:R12–4. 10.1016/s0960-9822(98)70008-9 9427622

[23] Opitz C, Soldati D. The glideosome: a dynamic complex powering gliding motion and host cell invasion by *toxoplasma gondii*: mechanism of host cell invasion by the Apicomplexa. Mol Microbiol. 2002;45:597–604. 10.1046/j.1365-2958.2002.03056.x 12139608

[24] Keeley A, Soldati D. The glideosome: a molecular machine powering motility and host-cell invasion by Apicomplexa. Trends Cell Biol. 2004;14:528–32. 10.1016/j.tcb.2004.08.002 15450974

[25] King C, Sleep J (2005). Modelling the mechanism of gregarine gliding using bead translocation. J Eukaryotic Microbiol.

[26] Frénal K, Dubremetz J-F, Lebrun M, Soldati-Favre D (2017). Gliding motility powers invasion and egress in Apicomplexa. Nat Rev Microbiol.

[27] Valigurová A, Vaškovicová N, Musilová N, Schrével J (2013). The enigma of eugregarine epicytic folds: where gliding motility originates?. Front Zool.

[28] Léger L, Duboscq O (1909). Etude sur la sexualité des Grégarines. Arch Protistenkd.

[29] Seppey M, Manni M, Zdobnov EM (2019). BUSCO: assessing genome assembly and annotation completeness. In gene prediction methods in molecular biology.

[30] Kumar S, Stecher G, Suleski M, Hedges SB. TimeTree: a resource for timelines, Timetrees, and divergence times. Mol Biol Evol. 2017;34:1812–9. 10.1093/molbev/msx116 28387841

[31] Cornillot E, Hadj-Kaddour K, Dassouli A, Noel B, Ranwez V, Vacherie B, et al. Sequencing of the smallest apicomplexan genome from the human pathogen *Babesia microti*. Nucleic Acids Res. 2012;40:9102–14. 10.1093/nar/gks700 PMC3467087 22833609

[32] Mathur V, Wakeman KC, Keeling PJ. Parallel functional reduction in the mitochondria of apicomplexan parasites. Curr Biol. 2021;31(13):2920-2928.e4. 10.1016/j.cub.2021.04.028 33974849

[33] Neafsey DE, Hartl DL, Berriman M. Evolution of noncoding and silent coding sites in the *Plasmodium falciparum* and *Plasmodium reichenowi* genomes. Mol Biol Evol. 2005;22:1621–6. 10.1093/molbev/msi154 15858207

[34] Reid AJ, Vermont SJ, Cotton JA, Harris D, Hill-Cawthorne GA, Könen-Waisman S, et al. Comparative genomics of the apicomplexan parasites *Toxoplasma gondii* and *Neospora caninum*: Coccidia differing in host range and transmission strategy. PLoS Pathog. 2012;8:e1002567. 10.1371/journal.ppat.1002567 PMC3310773 22457617

[35] Ricklefs RE, Outlaw DC. A molecular clock for malaria parasites. Science. 2010;329:226–9. 10.1126/science.1188954 20616281

[36] Hayakawa T, Tachibana S-I, Hikosaka K, Arisue N, Matsui A, Horii T, et al. Age of the last common ancestor of extant *Plasmodium* parasite lineages. Gene. 2012;502:36–9. 10.1016/j.gene.2012.04.037 22555021

[37] Crandall KA, Pérez-Losada M, Porter ML. Crabs, shrimps, and lobsters (Decapoda). In: The Timetree of life. New York: Oxford University Press; 2009. p. 551.

[38] Bracken-Grissom HD, Ahyong ST, Wilkinson RD, Feldmann RM, Schweitzer CE, Breinholt JW, et al. The emergence of lobsters: phylogenetic relationships, morphological evolution and divergence time comparisons of an ancient group (Decapoda: Achelata, Astacidea, Glypheidea, Polychelida). Syst Biol. 2014;63:457–79. 10.1093/sysbio/syu008 24562813

[39] Simdyanov TG, Diakin AY, Aleoshin VV. Ultrastructure and 28S rDNA phylogeny of two gregarines: *Cephaloidophora cf. communis* and *Heliospora *cf*. longissima* with remarks on gregarine morphology and phylogenetic analysis. Acta Protozool. 2015;54:241–62.

[40] Schrével J, Valigurová A, Prensier G, Chambouvet A, Florent I, Guillou L. Ultrastructure of *Selenidium pendula*, the type species of Archigregarines, and phylogenetic relations to other marine Apicomplexa. Protist. 2016;167:339–68. 10.1016/j.protis.2016.06.001 27423403

[41] Rueckert S, Simdyanov TG, Aleoshin VV, Leander BS. Identification of a divergent environmental DNA sequence clade using the phylogeny of gregarine parasites (Apicomplexa) from crustacean hosts. PLoS One. 2011;6:e18163. 10.1371/journal.pone.0018163 PMC3069048 21483868

[42] Mulec J, Summers Engel A. Karst spring microbial mat microeukaryotic diversity differs across an oxygen-sulphide ecocline and reveals potential for novel taxa discovery. AC. 2019;48.

[43] Skillman KM, Diraviyam K, Khan A, Tang K, Sept D, Sibley LD. Evolutionarily divergent, unstable filamentous actin is essential for gliding motility in apicomplexan parasites. PLoS Pathog. 2011;7:e1002280. 10.1371/journal.ppat.1002280 PMC3188518 21998582

[44] Plattner F, Yarovinsky F, Romero S, Didry D, Carlier M-F, Sher A, et al. *Toxoplasma* profilin is essential for host cell invasion and TLR11-dependent induction of an Interleukin-12 response. Cell Host Microbe. 2008;3:77–87. 10.1016/j.chom.2008.01.001 18312842

[45] Pino P, Sebastian S, Kim EA, Bush E, Brochet M, Volkmann K, et al. A tetracycline-repressible transactivator system to study essential genes in malaria parasites. Cell Host Microbe. 2012;12:824–34. 10.1016/j.chom.2012.10.016 PMC3712325 23245327

[46] Skillman KM, Daher W, Ma CI, Soldati-Favre D, Sibley LD. *Toxoplasma gondii* profilin acts primarily to sequester G-actin while Formins efficiently nucleate actin filament formation *in vitro*. Biochemistry. 2012;51:2486–95. 10.1021/bi201704y PMC3319672 22397711

[47] Mehta S, Sibley LD. Actin depolymerizing factor controls actin turnover and gliding motility in *Toxoplasma gondii*. MBoC. 2011;22:1290–9. 10.1091/mbc.E10-12-0939 PMC3078074 21346192

[48] Tosetti N, Pacheco NDS, Soldati-Favre D, Jacot D. Three F-actin assembly centers regulate organelle inheritance, cell-cell communication and motility in *Toxoplasma gondii*. Elife. 2019;12(8):e42669. 10.7554/eLife.42669 PMC6372287 30753127

[49] Daher W, Plattner F, Carlier M-F, Soldati-Favre D. Concerted action of two formins in gliding motility and host cell invasion by *toxoplasma gondii*. PLoS Pathog. 2010;6:e1001132. 10.1371/journal.ppat.1001132 PMC2951370 20949068

[50] Baum J, Tonkin CJ, Paul AS, Rug M, Smith BJ, Gould SB, et al. A malaria parasite Formin regulates actin polymerization and localizes to the parasite-erythrocyte moving junction during invasion. Cell Host Microbe. 2008;3:188–98. 10.1016/j.chom.2008.02.006 18329618

[51] Hunt A, Russell MRG, Wagener J, Kent R, Carmeille R, Peddie CJ, et al. Differential requirements for cyclase-associated protein (CAP) in actin-dependent processes of *Toxoplasma* gondii. eLife. 2019;8:e50598. 10.7554/eLife.50598 PMC6785269 31577230

[52] Ganter M, Schüler H, Matuschewski K (2009). Vital role for the *Plasmodium* actin capping protein (CP) beta-subunit in motility of malaria sporozoites. Mol Microbiol.

[53] Drewry LL, Sibley LD. *Toxoplasma* actin is required for efficient host cell invasion. mBio, 6. 2015:e00557–15. 10.1128/mBio.00557-15 PMC4471557 26081631

[54] Egarter S, Andenmatten N, Jackson AJ, Whitelaw JA, Pall G, Black JA, et al. The *Toxoplasma* Acto-MyoA motor complex is important but not essential for gliding motility and host cell invasion. PLoS One. 2014;9:e91819. 10.1371/journal.pone.0091819 PMC3954763 24632839

[55] Whitelaw JA, Latorre-Barragan F, Gras S, Pall GS, Leung JM, Heaslip A, et al. Surface attachment, promoted by the actomyosin system of *toxoplasma gondii* is important for efficient gliding motility and invasion. BMC Biol. 2017;15:1. 10.1186/s12915-016-0343-5 PMC5242020 28100223

[56] Meissner M, Schluter D, Soldati D. Role of *Toxoplasma gondii* myosin a in powering parasite gliding and host cell invasion. Science. 2002;298:837–40. 10.1126/science.1074553 12399593

[57] Frénal K, Marq J-B, Jacot D, Polonais V, Soldati-Favre D. Plasticity between MyoC- and MyoA-Glideosomes: An example of functional compensation in *Toxoplasma gondii* invasion. PLoS Pathog. 2014;10:e1004504. 10.1371/journal.ppat.1004504 PMC4231161 25393004

[58] Siden-Kiamos I, Ganter M, Kunze A, Hliscs M, Steinbüchel M, Mendoza J, et al. Stage-specific depletion of myosin a supports an essential role in motility of malarial ookinetes: promoter swap to study *Plasmodium* myosin a function. Cell Microbiol. 2011;13:1996–2006. 10.1111/j.1462-5822.2011.01686.x 21899701

[59] Bergman LW. Myosin a tail domain interacting protein (MTIP) localizes to the inner membrane complex of *Plasmodium* sporozoites. J Cell Sci. 2003;116:39–49. 10.1242/jcs.00194 12456714

[60] Gaskins E, Gilk S, DeVore N, Mann T, Ward G, Beckers C. Identification of the membrane receptor of a class XIV myosin in *Toxoplasma gondii*. J Cell Biol. 2004;165:383–93. 10.1083/jcb.200311137 PMC2172186 15123738

[61] Baum J, Papenfuss AT, Baum B, Speed TP, Cowman AF. Regulation of apicomplexan actin-based motility. Nat Rev Microbiol. 2006;4:621–8. 10.1038/nrmicro1465 16845432

[62] Frénal K, Polonais V, Marq J-B, Stratmann R, Limenitakis J, Soldati-Favre D. Functional dissection of the apicomplexan Glideosome molecular architecture. Cell Host Microbe. 2010;8:343–57. 10.1016/j.chom.2010.09.002 20951968

[63] Tardieux I, Baum J. Reassessing the mechanics of parasite motility and host-cell invasion. J Cell Biol. 2016;214:507–15. 10.1083/jcb.201605100 PMC5004448 27573462

[64] Bullen HE, Tonkin CJ, O’Donnell RA, Tham W-H, Papenfuss AT, Gould S, et al. A novel family of apicomplexan Glideosome-associated proteins with an inner membrane-anchoring role. J Biol Chem. 2009;284:25353–63. 10.1074/jbc.M109.036772 PMC2757237 19561073

[65] Graindorge A, Frénal K, Jacot D, Salamun J, Marq JB, Soldati-Favre D. The Conoid associated motor MyoH is indispensable for *Toxoplasma gondii* entry and exit from host cells. PLoS Pathog. 2016;12:e1005388. 10.1371/journal.ppat.1005388 PMC4711953 26760042

[66] Paing MM, Tolia NH. Multimeric assembly of host-pathogen adhesion complexes involved in apicomplexan invasion. PLoS Pathog. 2014;10:e1004120. 10.1371/journal.ppat.1004120 PMC4055764 24945143

[67] Boucher LE, Bosch J. The apicomplexan glideosome and adhesins – structures and function. J Struct Biol. 2015;190:93–114. 10.1016/j.jsb.2015.02.008 PMC4417069 25764948

[68] Jacot D, Waller RF, Soldati-Favre D, MacPherson DA, MacRae JI. Apicomplexan energy metabolism: carbon source promiscuity and the quiescence hyperbole. Trends Parasitol. 2016;32:56–70. 10.1016/j.pt.2015.09.001 26472327

[69] Sultan AA, Thathy V, Frevert U, Robson KJH, Crisanti A, Nussenzweig V, et al. TRAP is necessary for gliding motility and infectivity of *Plasmodium* Sporozoites. Cell. 1997;90:511–22. 10.1016/s0092-8674(00)80511-5 9267031

[70] Huynh M-H, Carruthers VB. *Toxoplasma* MIC2 is a major determinant of invasion and virulence. PLoS Pathog. 2006;2:e84. 10.1371/journal.ppat.0020084 PMC1550269 16933991

[71] Buguliskis JS, Brossier F, Shuman J, Sibley LD. Rhomboid 4 (ROM4) affects the processing of surface Adhesins and facilitates host cell invasion by *toxoplasma gondii*. PLoS Pathog. 2010;6(4):e1000858. 10.1371/journal.ppat.1000858 PMC2858701 20421941

[72] Shen B, Buguliskis JS, Lee TD, Sibley LD. Functional analysis of rhomboid proteases during *Toxoplasma* invasion. mBio, 5. 2014:e01795–14. 10.1128/mBio.01795-14 PMC4212836 25336455

[73] Rugarabamu G, Marq J-B, Guérin A, Lebrun M, Soldati-Favre D. Distinct contribution of *toxoplasma gondii* rhomboid proteases 4 and 5 to micronemal protein protease 1 activity during invasion: ROM4 and ROM5 contribute to MPP1 activity. Mol Microbiol. 2015;97:244–62. 10.1111/mmi.13021 25846828

[74] Kappe S, Bruderer T, Gantt S, Fujioka H, Nussenzweig V, Ménard R. Conservation of a gliding motility and cell invasion machinery in apicomplexan parasites. J Cell Biol. 1999;147:937–44. 10.1083/jcb.147.5.937 PMC2169348 10579715

[75] Morahan BJ, Wang L, Coppel RL. No TRAP, no invasion. Trends Parasitol. 2009;25:77–84. 10.1016/j.pt.2008.11.004 19101208

[76] Templeton TJ, Pain A. Diversity of extracellular proteins during the transition from the ‘proto-apicomplexan’ alveolates to the apicomplexan obligate parasites. Parasitology. 2016;143:1–17. 10.1017/S0031182015001213 26585326

[77] Dessens JT, Beetsma AL, Dimopoulos G, Wengelnik K, Crisanti A, Kafatos FC, et al. CTRP is essential for mosquito infection by malaria ookinetes. EMBO J. 1999;18:6221–7. 10.1093/emboj/18.22.6221 PMC1171685 10562534

[78] Bargieri DY. *Plasmodium* Merozoite TRAP family protein is essential for vacuole membrane disruption and gamete egress from erythrocytes. Cell Host Microbe. 2016;20:618–30. 10.1016/j.chom.2016.10.015 PMC5104695 27832590

[79] Lacroix C, Ménard R. TRAP-like protein of *Plasmodium* sporozoites: linking gliding motility to host-cell traversal. Trends Parasitol. 2008;24:431–4. 10.1016/j.pt.2008.07.003 18760672

[80] Deng M, Templeton TJ, London NR, Bauer C, Schroeder AA, Abrahamsen MS. *Cryptosporidium parvum* genes containing thrombospondin type 1 domains. IAI. 2002;70:6987–95. 10.1128/IAI.70.12.6987-6995.2002 PMC132954 12438378

[81] Putignani L, Possenti A, Cherchi S, Pozio E, Crisanti A, Spano F. The thrombospondin-related protein CpMIC1 (CpTSP8) belongs to the repertoire of micronemal proteins of *Cryptosporidium parvum*. Mol Biochem Parasitol. 2008;157:98–101. 10.1016/j.molbiopara.2007.09.004 17981348

[82] Gaffar FR, Yatsuda AP, Franssen FFJ, de Vries E. A *Babesia bovis* merozoite protein with a domain architecture highly similar to the thrombospondin-related anonymous protein (TRAP) present in *plasmodium* sporozoites. Mol Biochem Parasitol. 2004;136:25–34. 10.1016/j.molbiopara.2004.02.006 15138064

[83] Zhou J, Fukumoto S, Jia H, Yokoyama N, Zhang G, Fujisaki K, et al. Characterization of the *Babesia gibsoni* P18 as a homologue of thrombospondin related adhesive protein. Mol Biochem Parasitol. 2006;148:190–8. 10.1016/j.molbiopara.2006.03.015 16675041

[84] Yu L, Liu Q, Zhan X, Huang Y, Sun Y, Nie Z, et al. Identification and molecular characterization of a novel *Babesia orientalis* thrombospondin-related anonymous protein (BoTRAP1). Parasites Vectors. 2018;11:667. 10.1186/s13071-018-3245-2 PMC6307320 30587207

[85] Montenegro VN, Paoletta MS, Jaramillo Ortiz JM, Suarez CE, Wilkowsky SE. Identification and characterization of a *Babesia bigemina* thrombospondin-related superfamily member, TRAP-1: a novel antigen containing neutralizing epitopes involved in merozoite invasion. Parasites Vectors. 2020;13:602. 10.1186/s13071-020-04469-5 PMC7705850 33261638

[86] Lovett J. Molecular characterization of a thrombospondin-related anonymous protein homologue in *Neospora caninum*. Mol Biochem Parasitol. 2000;107:33–43. 10.1016/s0166-6851(99)00228-5 10717300

[87] Clarke LE, Tomley FM, Wisher MH, Foulds IJ, Boursnell ME. Regions of an *Eimeria tenella* antigen contain sequences which are conserved in circumsporozoite proteins from *plasmodium* spp. and which are related to the thrombospondin gene family. Mol Biochem Parasitol. 1990;41:269–79. 10.1016/0166-6851(90)90190-w 2204833

[88] Witcombe DM, Belli SI, Wallach MG, Smith NC. Molecular characterisation of EmTFP250: a novel member of the TRAP protein family in *Eimeria maxima*. Int J Parasitol. 2003;33:691–702. 10.1016/s0020-7519(03)00086-9 12814649

[89] Bichet M, Joly C, Hadj Henni A, Guilbert T, Xémard M, Tafani V, et al. The *Toxoplasma*-host cell junction is anchored to the cell cortex to sustain parasite invasive force. BMC Biol. 2014;12:773. 10.1186/s12915-014-0108-y PMC4316648 25551479

[90] Portes J, Barrias E, Travassos R, Attias M, de Souza W. *Toxoplasma gondii* mechanisms of entry into host cells. Front Cell Infect Microbiol. 2020;10:294. 10.3389/fcimb.2020.00294 PMC7340009 32714877

[91] Yang ASP, Lopaticki S, O’Neill MT, Erickson SM, Douglas DN, Kneteman NM, et al. AMA1 and MAEBL are important for *plasmodium falciparum* sporozoite infection of the liver. Cell Microbiol. 2017;19:e12745. 10.1111/cmi.12745 28371168

[92] O’Hara SP, Chen X-M. The cell biology of *Cryptosporidium* infection. Microbes Infect. 2011;13:721–30. 10.1016/j.micinf.2011.03.008 PMC3130844 21458585

[93] Singh P, Mirdha BR, Srinivasan A, Rukmangadachar LA, Singh S, Sharma P, et al. Identification of invasion proteins of *Cryptosporidium parvum*. World J Microbiol Biotechnol. 2015;31:1923–34. 10.1007/s11274-015-1936-9 26492887

[94] Lourido S, Moreno SNJ. The calcium signaling toolkit of the apicomplexan parasites *Toxoplasma gondii* and *Plasmodium* spp. Cell Calcium. 2015;57:186–93. 10.1016/j.ceca.2014.12.010 PMC4428288 25605521

[95] Ghartey-Kwansah G, Yin Q, Li Z, Gumpper K, Sun Y, Yang R, et al. Calcium-dependent protein kinases in malaria parasite development and infection. Cell Transplant. 2020;29:096368971988488. 10.1177/0963689719884888 PMC7444236 32180432

[96] Bullen HE, Jia Y, Yamaryo-Botté Y, Bisio H, Zhang O, Jemelin NK, et al. Phosphatidic acid-mediated signaling regulates Microneme secretion in *Toxoplasma*. Cell Host Microbe. 2016;19:349–60. 10.1016/j.chom.2016.02.006 26962945

[97] Darvill N, Dubois DJ, Rouse SL, Hammoudi P-M, Blake T, Benjamin S, et al. Structural basis of phosphatidic acid sensing by APH in apicomplexan parasites. Structure. 2018;26:1059–71. 10.1016/j.str.2018.05.001 PMC6084407 29910186

[98] Farrell A, Thirugnanam S, Lorestani A, Dvorin JD, Eidell KP, Ferguson DJP, et al. A DOC2 protein identified by mutational profiling is essential for apicomplexan parasite exocytosis. Science. 2012;335:218–21. 10.1126/science.1210829 PMC3354045 22246776

[99] Heaslip AT, Nishi M, Stein B, Hu K. The motility of a human parasite, *Toxoplasma gondii*, is regulated by a novel lysine methyltransferase. PLoS Pathog. 2011;7:e1002201. 10.1371/journal.ppat.1002201 PMC3164638 21909263

[100] Piganeau G, Eyre-Walker A, Grimsley N, Moreau H. How and why DNA barcodes underestimate the diversity of microbial eukaryotes. PLoS One. 2011;6:e16342. 10.1371/journal.pone.0016342 PMC3037371 21347361

[101] Guo Y, Tang K, Rowe LA, Li N, Roellig DM, Knipe K, et al. Comparative genomic analysis reveals occurrence of genetic recombination in virulent *Cryptosporidium hominis* subtypes and telomeric gene duplications in *Cryptosporidium parvum*. BMC Genomics. 2015;16:320. 10.1186/s12864-015-1517-1 PMC4407392 25903370

[102] Gras S, Jackson A, Woods S, Pall G, Whitelaw J, Leung JM, et al. Parasites lacking the micronemal protein MIC2 are deficient in surface attachment and host cell egress, but remain virulent in vivo. Wellcome Open Res. 2017;2:32. 10.12688/wellcomeopenres.11594.2 PMC5473411 28630943

[103] Harding CR, Gow M, Kang JH, Shortt E, Manalis SR, Meissner M, et al. Alveolar proteins stabilize cortical microtubules in *Toxoplasma gondii*. Nature. Communications. 2019;10:401. 10.1038/s41467-019-08318-7 PMC6344517 30674885

[104] Schlott AC, Knuepfer E, Green JL, Hobson P, Borg AJ, Morales-Sanfrutos J, et al. Inhibition of protein N-myristoylation blocks *Plasmodium falciparum* intraerythrocytic development, egress and invasion. PLoS Biol. 2021;19:e3001408. 10.1371/journal.pbio.3001408 PMC8544853 34695132

[105] Rompikuntal PK, Kent RS, Foe IT, Deng B, Bogyo M, Ward GE. Blocking palmitoylation of *Toxoplasma gondii* myosin light chain 1 disrupts glideosome composition but has little impact on parasite motility. mSphere. 2021;6(3):e00823-20. 10.1128/mSphere.00823-20 PMC8265671 34011689

[106] Valigurová A, Vaškovicová N, Diakin A, Paskerova GG, Simdyanov TG, Kováčiková M. Motility in blastogregarines (Apicomplexa): native and drug-induced organisation of *Siedleckia nematoides* cytoskeletal elements. PLoS One. 2017;12:e0179709. 10.1371/journal.pone.0179709 PMC5480980 28640849

[107] Heintzelman MB. Actin and myosin in *Gregarina polymorpha*. Cell Motil Cytoskeleton. 2004;58:83–95. 10.1002/cm.10178 15083530

[108] Heintzelman MB, Mateer MJ. GpMyoF, a WD40 repeat-containing myosin associated with the Myonemes of *Gregarina polymorpha*. J Parasitol. 2008;94:158–68. 10.1645/GE-1339.1 18372636

[109] Kováčiková M, Simdyanov TG, Diakin A, Valigurová A. Structures related to attachment and motility in the marine eugregarine *Cephaloidophora *cf.* communis* (Apicomplexa). Eur J Protistol. 2017;59:1–13. 10.1016/j.ejop.2017.02.006 28363137

[110] Diakin A, Wakeman KC, Valigurová A (2017). Description of *Ganymedes yurii* sp. n. (Ganymedidae), a new gregarine species from the Antarctic amphipod *Gondogeneia* sp. (Crustacea). J Eukaryot Microbiol.

[111] Butler M, Cockcroft A, MacDiarmid A, Wahle R. *Homarus gammarus*. The IUCN Red List of Threatened Species. 2011:e.T169955A69905303. http://www.iucnredlist.org/details/169955/0. Accessed 27 June 2022.

[112] Andrews S. FastQC: a quality control tool for high throughput sequence data. 2010. https://www.bioinformatics.babraham.ac.uk/projects/fastqc/. Accessed 27 June 2022.

[113] Krueger F. Trim galore. A wrapper tool around Cutadapt and FastQC to consistently apply quality and adapter trimming to FastQ files. 2015. https://github.com/FelixKrueger/TrimGalore. Accessed 27 June 2022.

[114] Bankevich A, Nurk S, Antipov D, Gurevich AA, Dvorkin M, Kulikov AS, et al. SPAdes: a new genome assembly algorithm and its applications to single-cell sequencing. J Comput Biol. 2012;19:455–77. 10.1089/cmb.2012.0021 PMC3342519 22506599

[115] Grabherr MG, Haas BJ, Yassour M, Levin JZ, Thompson DA, Amit I, et al. Full-length transcriptome assembly from RNA-Seq data without a reference genome. Nat Biotechnol. 2011;29:644–52. 10.1038/nbt.1883 PMC3571712 21572440

[116] Haas BJ, Papanicolaou A, Yassour M, Grabherr M, Blood PD, Bowden J, et al. De novo transcript sequence reconstruction from RNA-seq using the trinity platform for reference generation and analysis. Nat Protoc. 2013;8:1494–512. 10.1038/nprot.2013.084 PMC3875132 23845962

[117] Stanke M, Keller O, Gunduz I, Hayes A, Waack S, Morgenstern B. AUGUSTUS: *ab initio* prediction of alternative transcripts. Nucleic Acids Res. 2006;34:W435–9. 10.1093/nar/gkl200 PMC1538822 16845043

[118] Altschul SF, Gish W, Miller W, Myers EW, Lipman DJ. Basic local alignment search tool. J Mol Biol. 1990;215:403–10. 10.1016/S0022-2836(05)80360-2 2231712

[119] Langmead B, Salzberg SL. Fast gapped-read alignment with bowtie 2. Nat Methods. 2012;9:357–9. 10.1038/nmeth.1923 PMC3322381 22388286

[120] Li H, Handsaker B, Wysoker A, Fennell T, Ruan J, Homer N, et al. The sequence alignment/map format and SAMtools. Bioinformatics. 2009;25:2078–9. 10.1093/bioinformatics/btp352 PMC2723002 19505943

[121] Quinlan AR, Hall IM. BEDTools: a flexible suite of utilities for comparing genomic features. Bioinformatics. 2010;26:841–2. 10.1093/bioinformatics/btq033 PMC2832824 20110278

[122] Kurtz S, Phillippy A, Delcher AL, Smoot M, Shumway M, Antonescu C, et al. Versatile and open software for comparing large genomes. Genome Biol. 2004;5:R12. 10.1186/gb-2004-5-2-r12 PMC395750 14759262

[123] Gurevich A, Saveliev V, Vyahhi N, Tesler G. QUAST: quality assessment tool for genome assemblies. Bioinformatics. 2013;29:1072–5. 10.1093/bioinformatics/btt086 PMC3624806 23422339

[124] Wu TD, Watanabe CK. GMAP: a genomic mapping and alignment program for mRNA and EST sequences. Bioinformatics. 2005;21:1859–75. 10.1093/bioinformatics/bti310 15728110

[125] Haas BJ. Improving the *Arabidopsis* genome annotation using maximal transcript alignment assemblies. Nucleic Acids Res. 2003;31:5654–66. 10.1093/nar/gkg770 PMC206470 14500829

[126] Korf I. Gene finding in novel genomes. BMC Bioinformatics. 2004;5:59. 10.1186/1471-2105-5-59 PMC421630 15144565

[127] Smit Hubley, and Green. RepeatMasker Open-4.0. 2015. http://www.repeatmasker.org. Accessed 27 June 2022.

[128] Aramaki T, Blanc-Mathieu R, Endo H, Ohkubo K, Kanehisa M, Goto S, et al. KofamKOALA: KEGG Ortholog assignment based on profile HMM and adaptive score threshold. Bioinformatics. 2020;36:2251–2. 10.1093/bioinformatics/btz859 PMC7141845 31742321

[129] Jones P, Binns D, Chang H-Y, Fraser M, Li W, McAnulla C, et al. InterProScan 5: genome-scale protein function classification. Bioinformatics. 2014;30:1236–40. 10.1093/bioinformatics/btu031 PMC3998142 24451626

[130] Li, L., Stoeckert, CJ., and Roos, D. (2003). OrthoMCL: identification of Ortholog groups for eukaryotic genomes. Genome Res 13, 2178–2189. 10.1101/gr.1224503 PMC403725 12952885

[131] Ranwez V, Harispe S, Delsuc F, Douzery EJP. MACSE: multiple alignment of coding SEquences accounting for frameshifts and stop codons. PLoS One. 2011;6:e22594. 10.1371/journal.pone.0022594 PMC3174933 21949676

[132] Yang Z, Nielsen R. Estimating synonymous and nonsynonymous substitution rates under realistic evolutionary models. Mol Biol Evol. 2000;17:32–43. 10.1093/oxfordjournals.molbev.a026236 10666704

[133] Yang Z. PAML 4: phylogenetic analysis by maximum likelihood. Mol Biol Evol. 2007;24:1586–91. 10.1093/molbev/msm088 17483113

[134] Nawrocki EP, Eddy SR. Infernal 1.1: 100-fold faster RNA homology searches. Bioinformatics. 2013;29:2933–5. 10.1093/bioinformatics/btt509 PMC3810854 24008419

[135] Burge SW, Daub J, Eberhardt R, Tate J, Barquist L, Nawrocki EP, et al. Rfam 11.0: 10 years of RNA families. Nucleic Acids Res. 2013;41:D226–32. 10.1093/nar/gks1005 PMC3531072 23125362

[136] de Araujo Oliveira JV, Costa F, Backofen R, Stadler PF, Walter MT, M.E., and Hertel, J. SnoReport 2.0: new features and a refined support vector machine to improve snoRNA identification. BMC Bioinformatics. 2016;17:464. 10.1186/s12859-016-1345-6 PMC5249026 28105919

[137] Karadjian G, Hassanin A, Saintpierre B, Gembu Tungaluna G-C, Ariey F, Ayala FJ, et al. Highly rearranged mitochondrial genome in *Nycteria* parasites (Haemosporidia) from bats. Proc Natl Acad Sci. 2016;113:9834–9. 10.1073/pnas.1610643113 PMC5024609 27528689

[138] Katoh K, Standley DM. MAFFT multiple sequence alignment software version 7: improvements in performance and usability. Mol Biol Evol. 2013;30:772–80. 10.1093/molbev/mst010 PMC3603318 23329690

[139] Stamatakis A. RAxML version 8: a tool for phylogenetic analysis and post-analysis of large phylogenies. Bioinformatics. 2014;30:1312–3. 10.1093/bioinformatics/btu033 PMC3998144 24451623

[140] Whelan S, Irisarri I, Burki F. PREQUAL: detecting non-homologous characters in sets of unaligned homologous sequences. Bioinformatics. 2018;34:3929–30. 10.1093/bioinformatics/bty448 29868763

[141] Ali RH, Bogusz M, Whelan S. Identifying clusters of high confidence homologies in multiple sequence alignments. Mol Biol Evol. 2019;36:2340–51. 10.1093/molbev/msz142 PMC6933875 31209473

[142] Capella-Gutiérrez S, Silla-Martínez JM, Gabaldón T. trimAl: a tool for automated alignment trimming in large-scale phylogenetic analyses. Bioinformatics. 2009;25:1972–3. 10.1093/bioinformatics/btp348 PMC2712344 19505945

[143] Tice AK, Žihala D, Pánek T, Jones RE, Salomaki ED, Nenarokov S, Burki F, Eliáš M, Eme L, Roger AJ (2021). PhyloFisher: a phylogenomic package for resolving eukaryotic relationships. PLoS Biol.

[144] Minh BQ, Schmidt HA, Chernomor O, Schrempf D, Woodhams MD, von Haeseler A, Lanfear R (2020). IQ-TREE 2: new models and efficient methods for phylogenetic inference in the genomic era. Mol Biol Evol.

[145] Ronquist F, Huelsenbeck JP (2003). MrBayes 3: Bayesian phylogenetic inference under mixed models. Bioinformatics.

[146] Rambaut (2018). FigTree. tree.bio.ed.ac.uk/software/figtree/.

[147] Woo YH, Ansari H, Otto TD, Klinger CM, Kolisko M, Michálek J, et al. Chromerid genomes reveal the evolutionary path from photosynthetic algae to obligate intracellular parasites. eLife. 2015;4:e06974. 10.7554/eLife.06974 PMC4501334 26175406

[148] Castresana J (2000). Selection of conserved blocks from multiple alignments for their use in phylogenetic analysis. Mol Biol Evol.

[149] Kumar S, Stecher G, Li M, Knyaz C, Tamura K (2018). MEGA X: molecular evolutionary genetics analysis across computing platforms. Mol Biol Evol.

[150] Letunic I, Khedkar S, Bork P (2021). SMART: recent updates, new developments and status in 2020. Nucleic Acids Res.

[151] Wickham H. Ggplot2: elegant graphics for data analysis. New York: Springer; 2009.

[152] Birney E (2004). GeneWise and Genomewise. Genome Res.

