# Supplementary material for: Marine gregarine genomes reveal the breadth of apicomplexan diversity with a partially conserved glideosome machinery
Source: BMC Genomics. 2022 Jul 2;23:485. doi: 10.1186/s12864-022-08700-8 (PMC9250747; doi:10.1186/s12864-022-08700-8)
Supplement: Supplementary file 2 — Additional file 2. [file 12864_2022_8700_MOESM2_ESM.pdf]

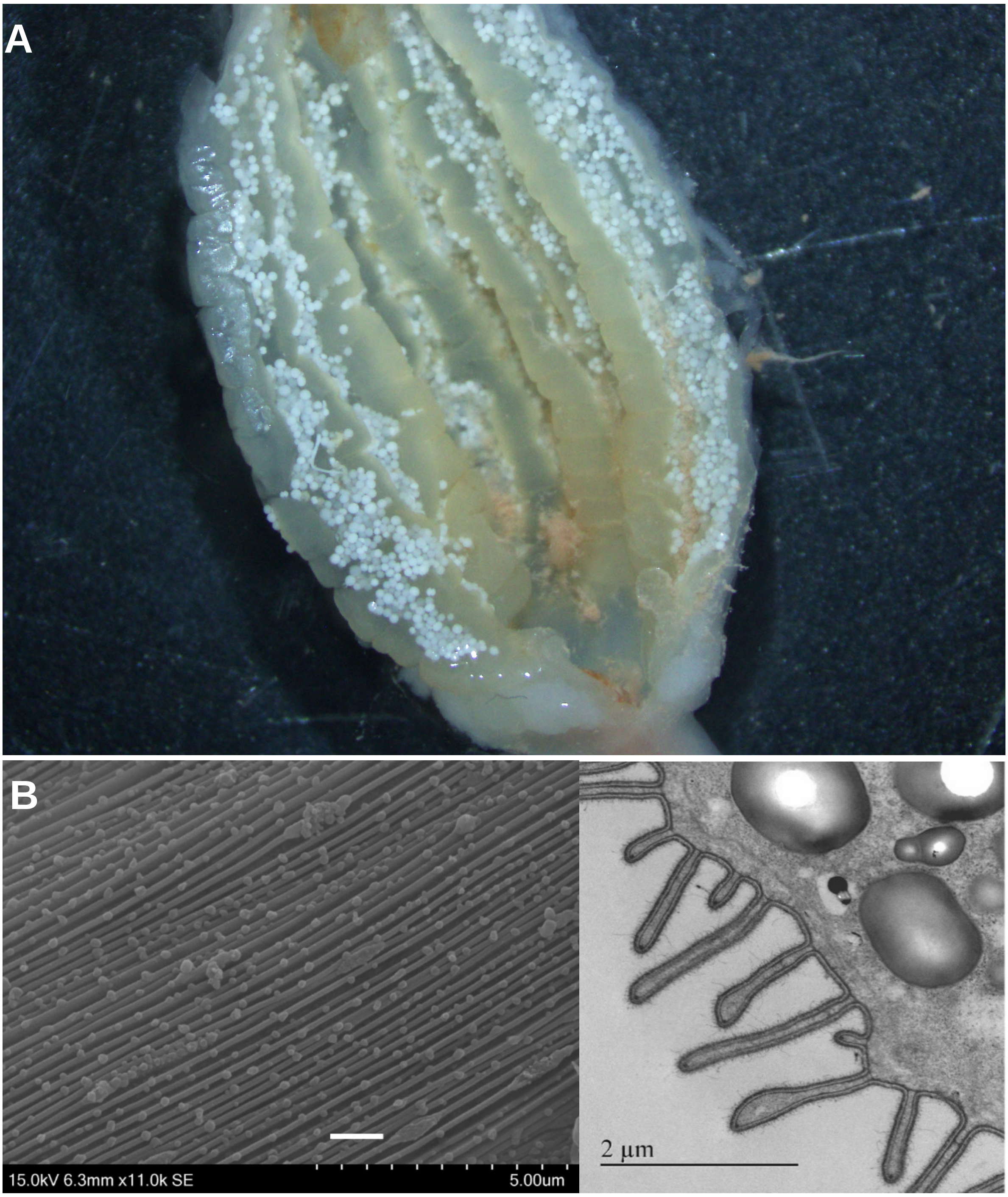

Figure S1. Additional microscopy figures, related to Figure 1. A. Photonic image of the rectal ampulla of Lobster#12, longitudinally opened and heavily packed with *Porospora* cf. *gigantea* cysts in chitinous folds. The length of the rectal ampulla is about 3 cm. B. Morphological evidence for epicytic folds. Zoom on epicytic folds for trophozoite#9, Lobster#12. Scale=1µm. SEM imaging (left); TEM imaging (right).

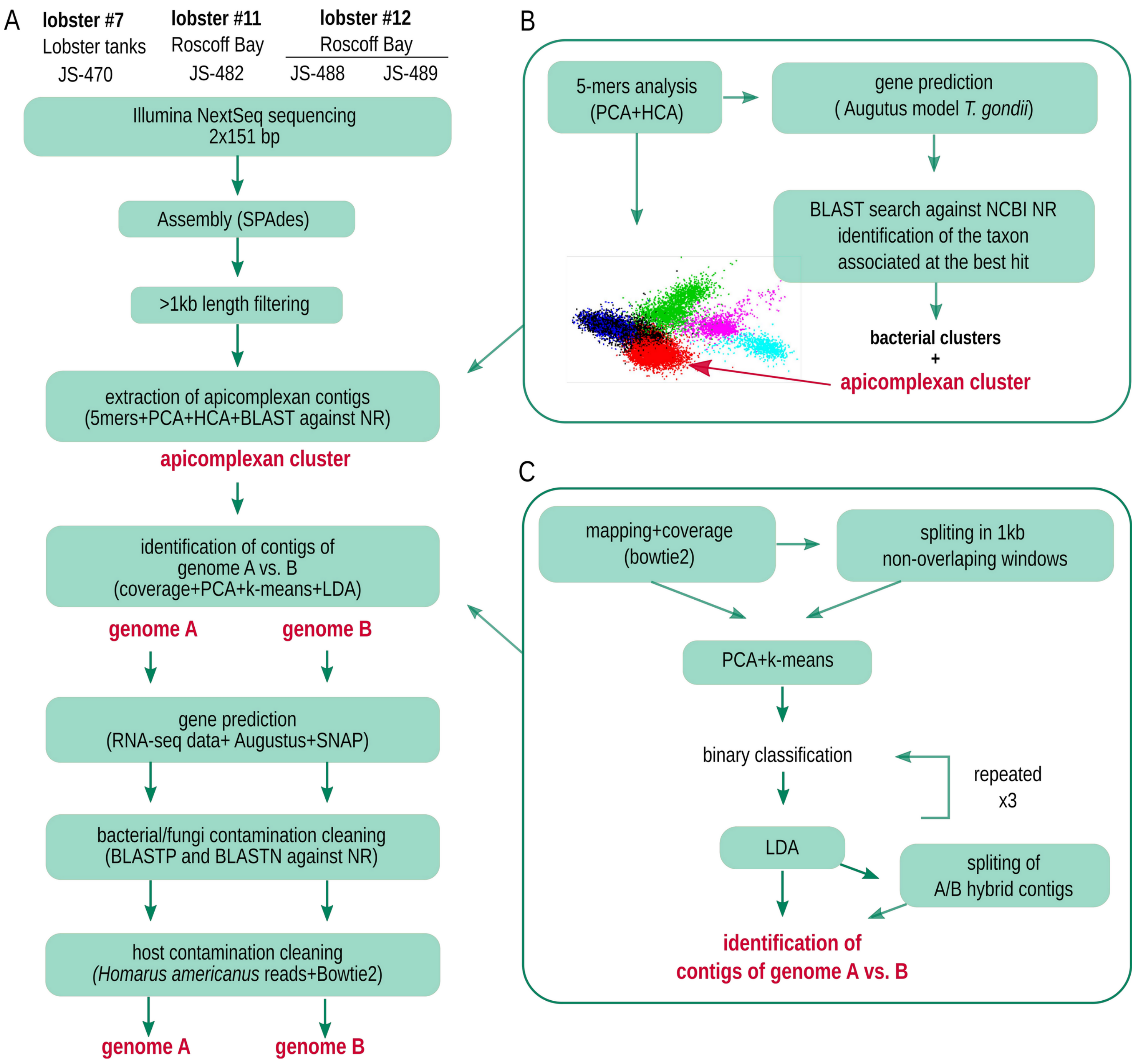

Figure S2. Protocol for assembling the two genomes. A. Overview of the full protocol. B. Identification of apicomplexan vs contaminant contigs based on k-mer composition. C. Identification of contigs from genomes A and B based on coverage data for each individual library. See also Figures S3, S4 and S5.

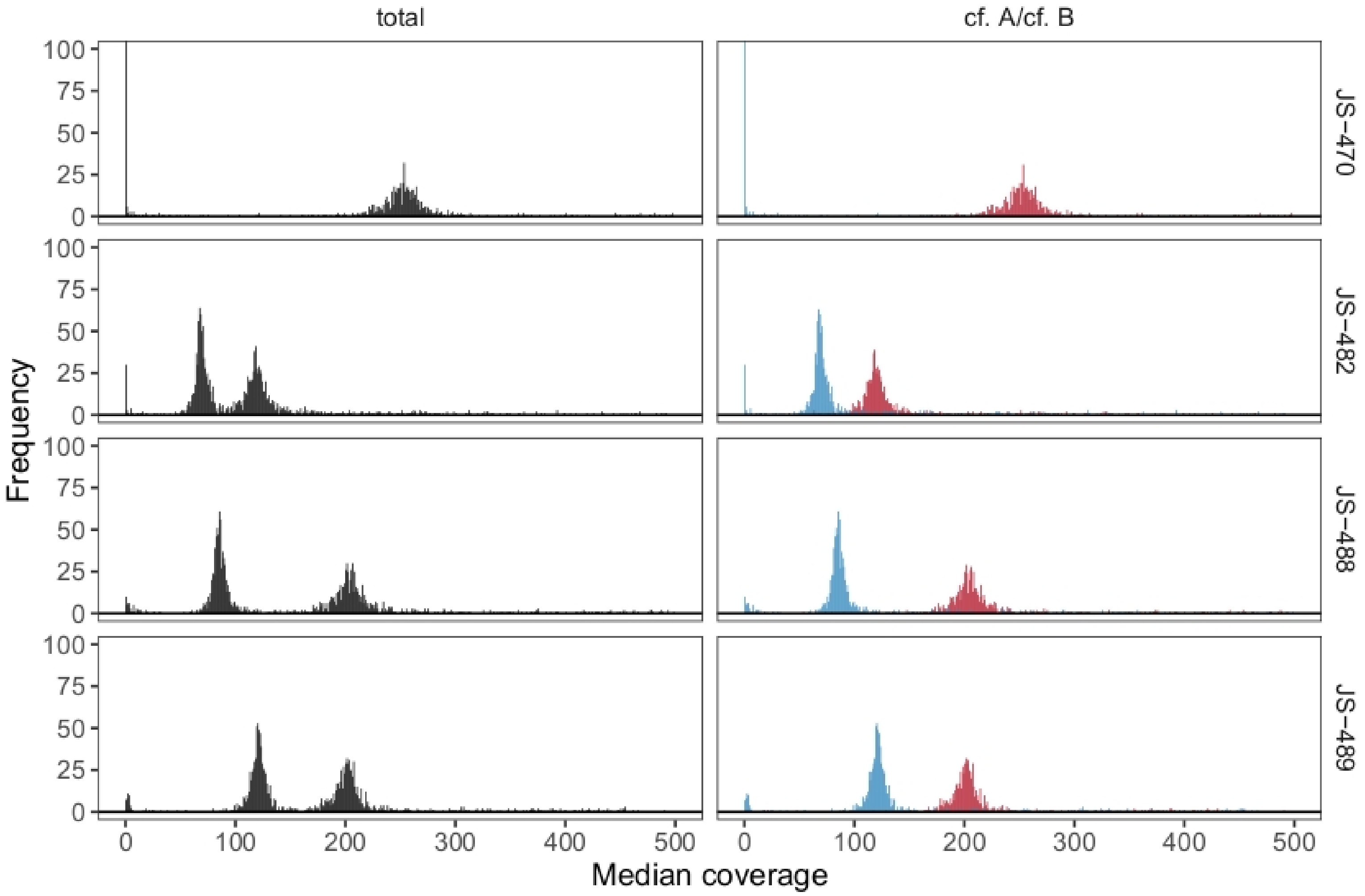

Figure S3. Distribution of the median coverage in each individual library calculated for each contig from the raw assembly, related to Figure S2. Total coverage are presented in black (left side). After genomic attribution of each contig, plot is presented again in red for *Porospora* cf. *gigantea* A and in blue for *Porospora* cf. *gigantea* B (right side).

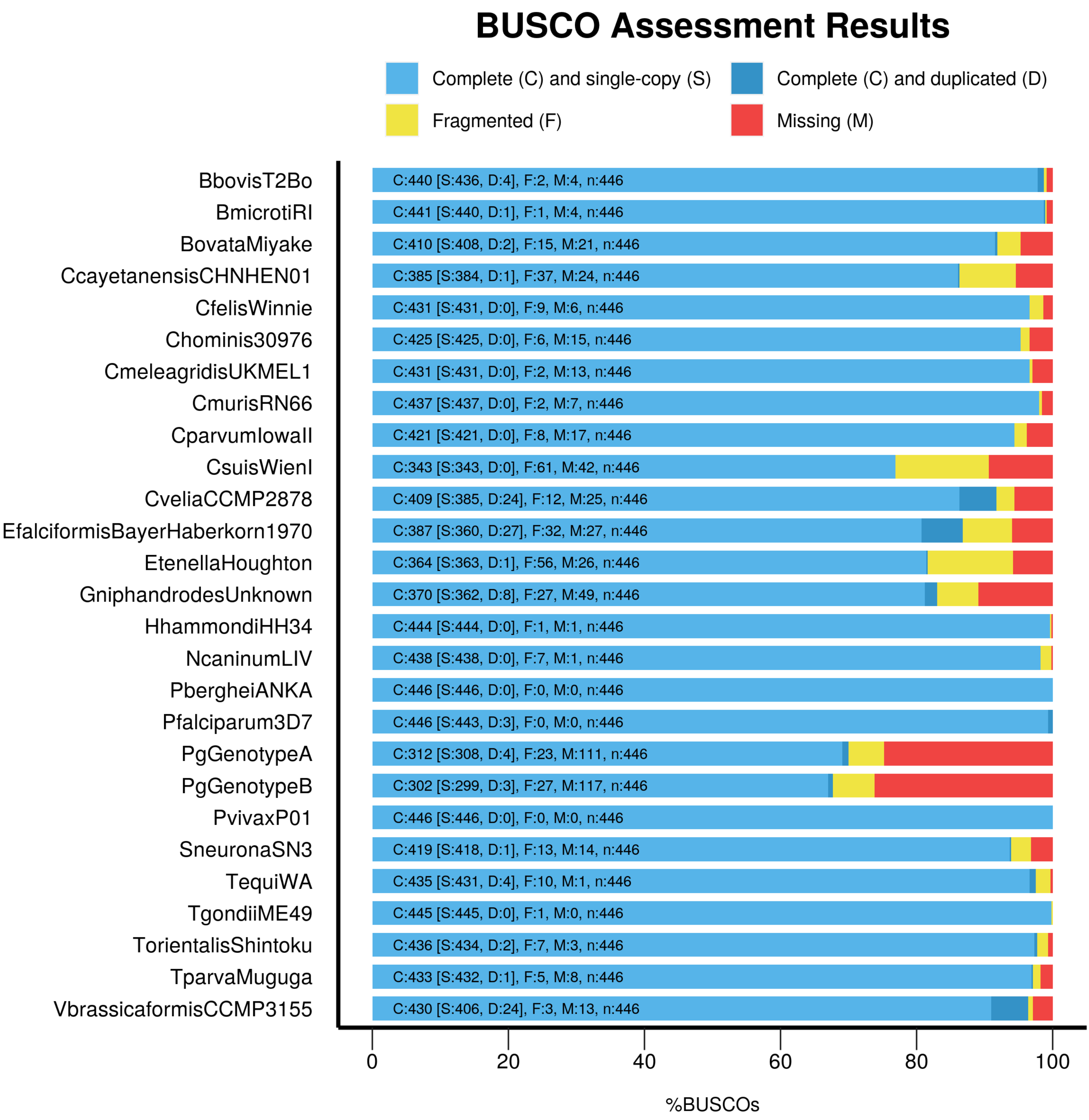

Figure S4. BUSCOs assessment results for the proteomes of both *P.* cf. *gigantea* and a selection of 25 reference species (geneset apicomplexa_odb10), Related to Figure S2.

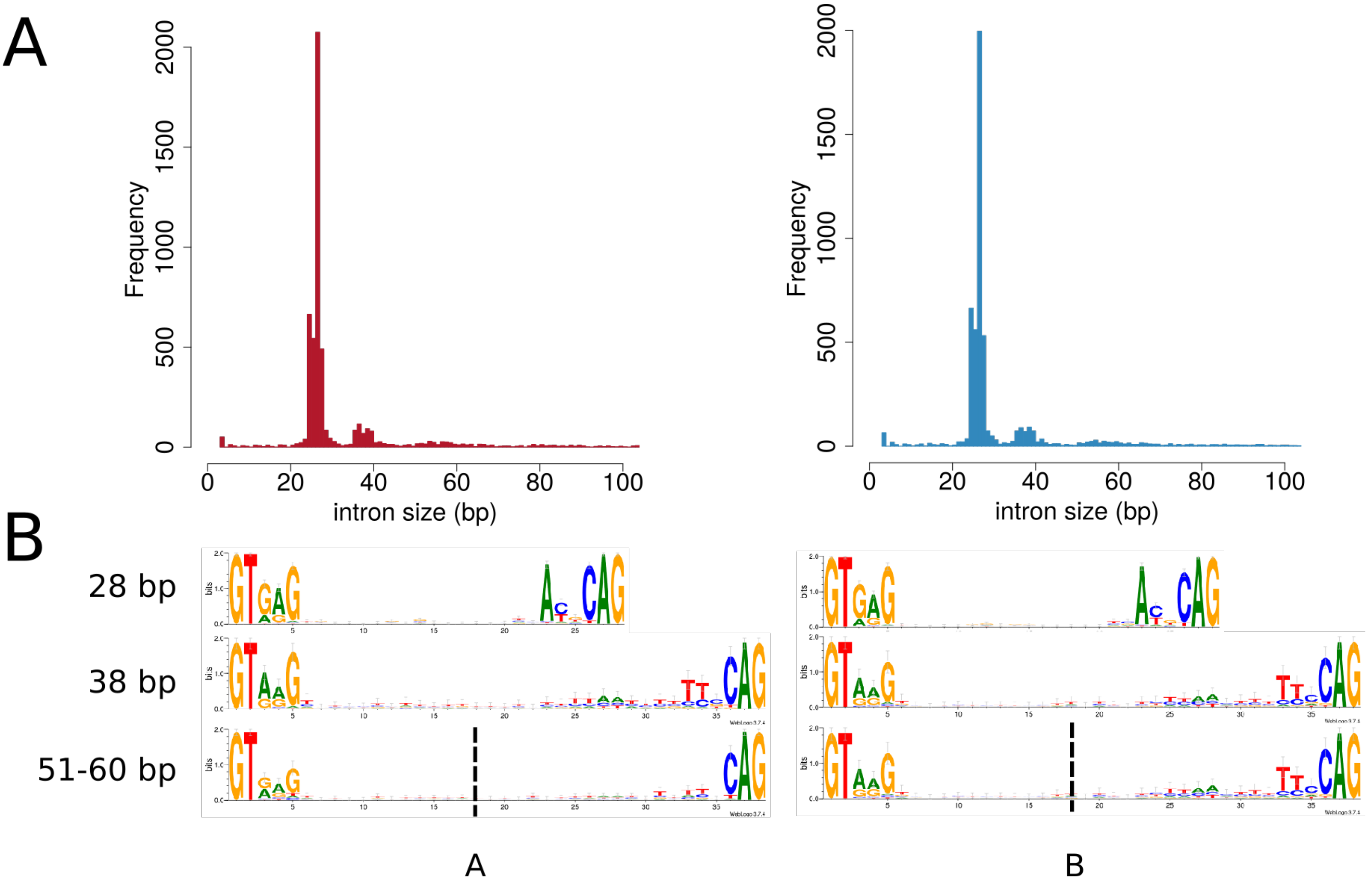

Figure S5. Introns of both *P.* cf. *gigantea* genomes, related to Table 1 and Figure S2. A. Length distribution B. Consensus for the major class of short introns (28bp long) and the two other alternative classes (38bp long and range from 51 to 60bp). For 51-60bp introns, donor sites are presented by 17 first nt, acceptor sites are presented by the 19 last nt, separated by dashes. Data for *P.* cf. *gigantea* A (left side) and B (right side).

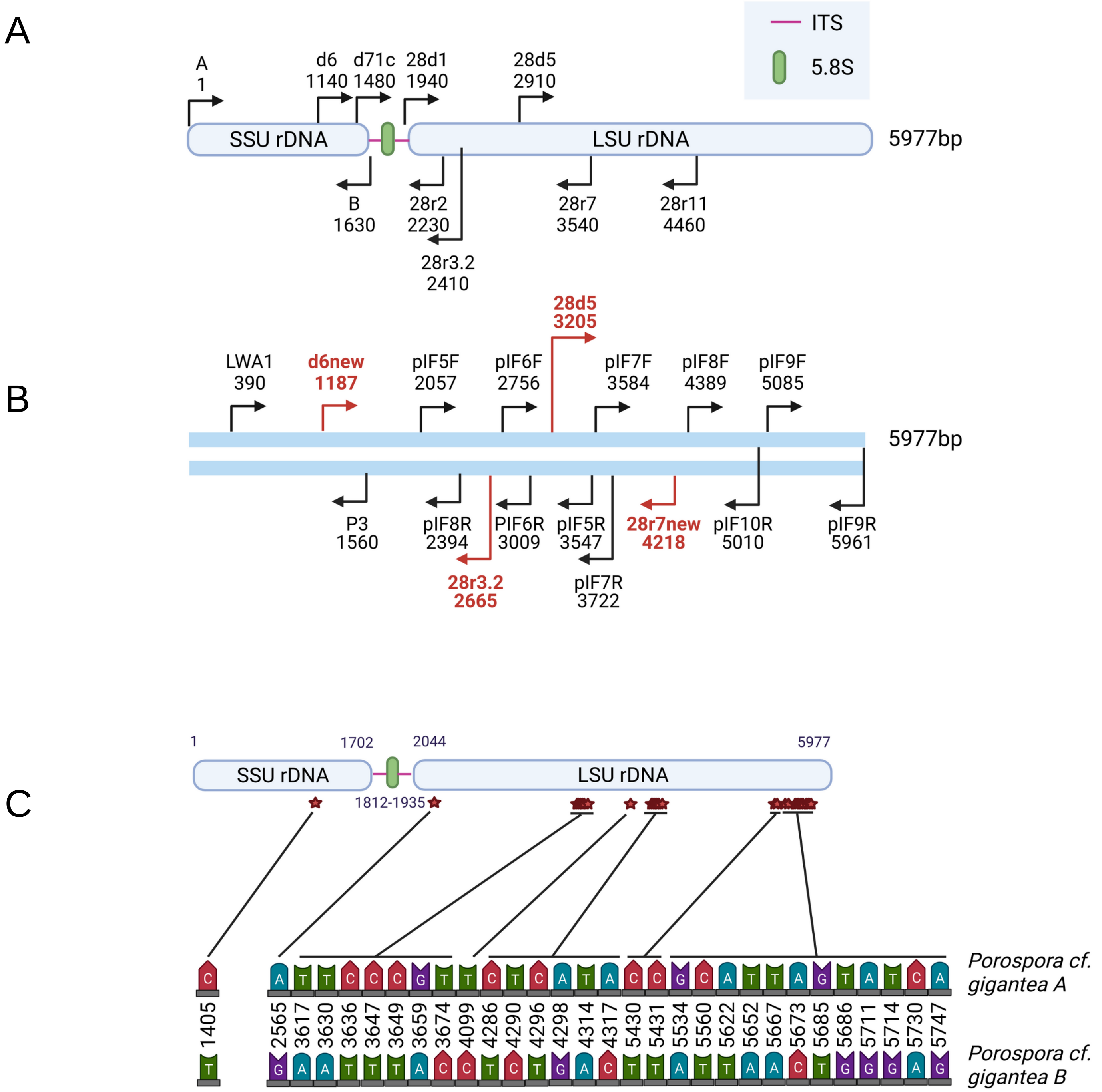

Figure S6. Complete ribosomal locus reconstruction for *Porospora* cf. *gigantea*, related to Figure S2. A. Complete ribosomal locus for *Cephaloidophora cf. communis* and *Heliospora* cf. *longissima* from Simdyanov et al. (2015)[S1]. B. Complete ribosomal locus for *Porospora* cf. *gigantea* A using primers based on Simdyanov et al. (2015)[S1] (red) and novel primers (black) to experimentally amplify and sequence the complete 5977bp locus. See also supp. Table 3 for primer sequences. C. Distribution of the 30 polymorphic positions between A and B complete ribosomal loci.

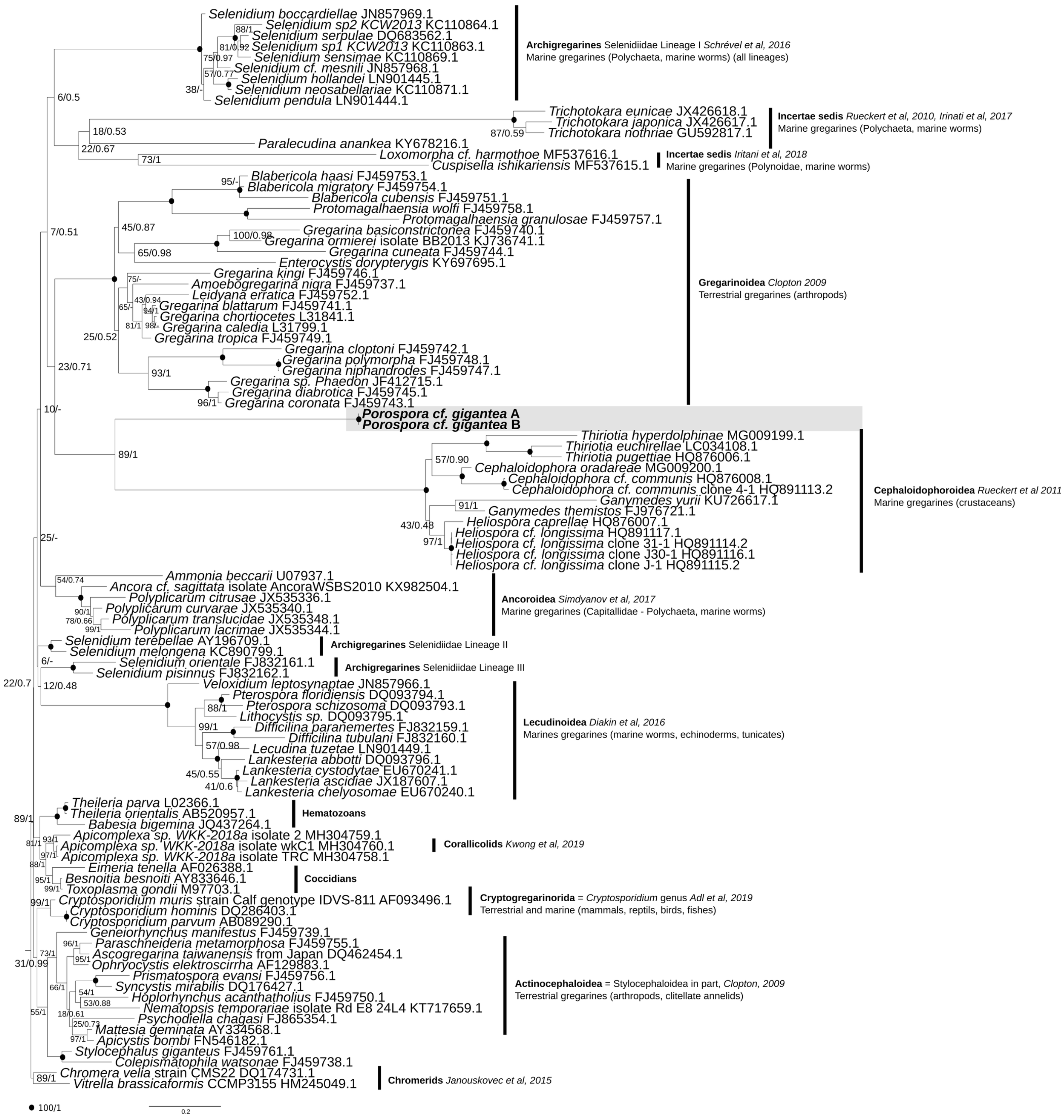

Figure S7. Gregarines/apicomplexan phylogeny, Related to Figure 3. Phylogenetic tree built using 100 18S rDNA sequences 1614 sites in order to situate *P.* cf. *gigantea* A and B among other known gregarines and apicomplexan clades. Chromerid sequences were used as outgroup, as they are considered as the sister group of all other apicomplexans[S2]. Evolutionary history was inferred by maximum likelihood and bayesian inference using a GTR+G+I model. Topologies were identical according to both methods. Black spots indicate 100/1 supports. Supports <70/0.7 are not shown. Families and associated literature are indicated.

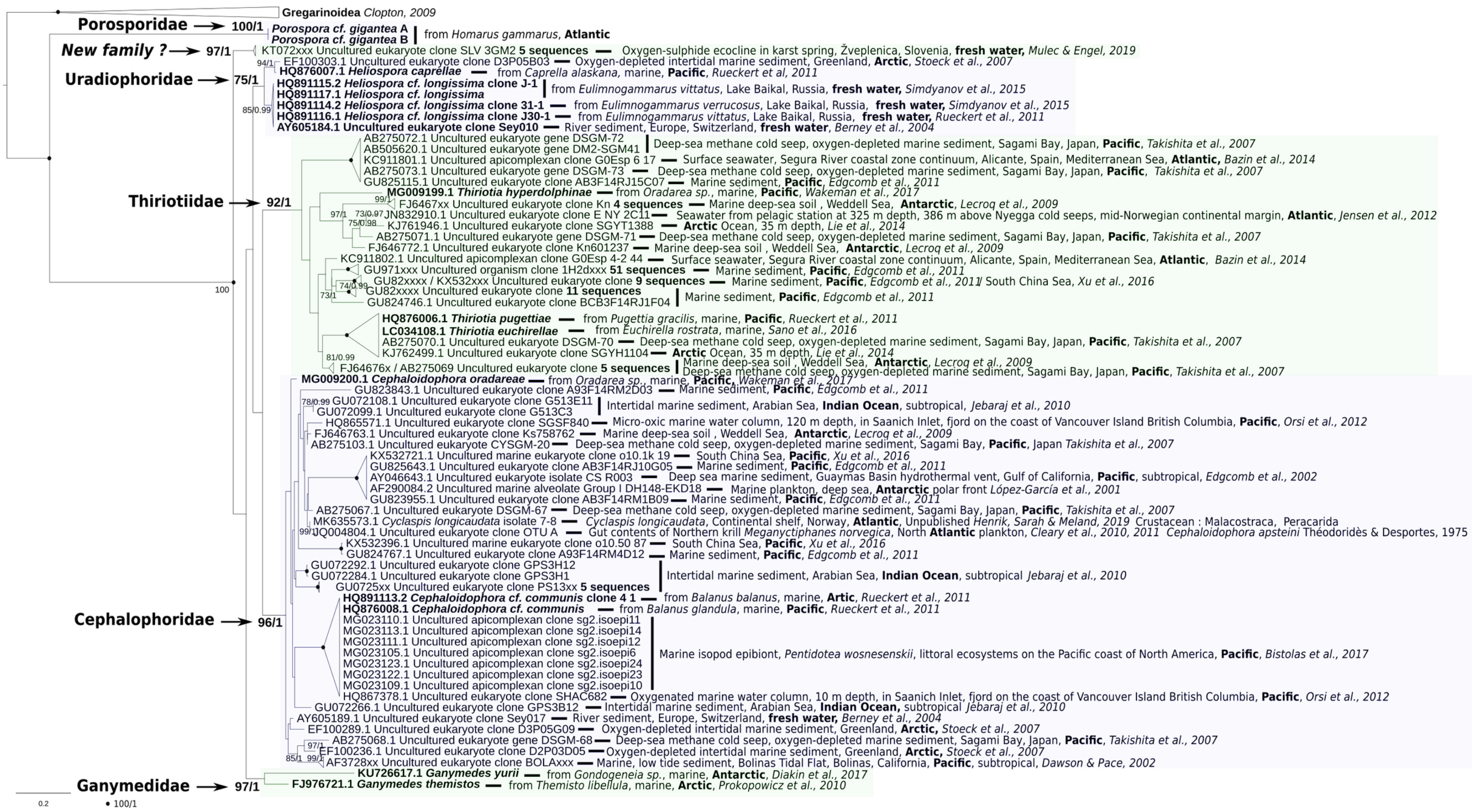

Figure S8. Environmental phylogeny, related to Figure 3. Phylogenetic tree built using 189 18S rDNA sequences for 1135 sites in order to situate two *P.* cf. *gigantea* A and B among other crustacean gregarines and environmental sequences. Considering that Gregarinoidea sequences were placed as sister group of other crustaceans' gregarines in the gregarines/apicomplexan phylogeny, as well as in recent literature[S3,S4,S5], they were used as outgroup. Evolutionary history was inferred by maximum likelihood and bayesian inference using a GTR+G+I model. Topologies are identical according to both methods. Black spots indicate 100/1 supports. Supports <70/0.7 are not shown. Geographical provenance of all environmental sequences are indicated and their localization is highlighted in bold.

Species	Strain	Gene count (a)	Contigs (a)	Total length (Mb)(b)	GC (%) (b)	Publication (a)
Cryptosporidium hominis	30976	3994	53	9.059	30.13	Guo et al, 2016[S6]
Cryptosporidium muris	RN66	3981	75	9.242	28.47	x
Cryptosporidium meleagridis	UKMEL1	3806	57	8.973	30.97	Ifeonu et al, 2016[S7]
**Cryptosporidium parvum (1)**	**IowaII**	**4020**	**8**	**9.102**	**30.22**	**Abramhasen et al, 2004**[S8]
**Chromera velia (1)**	**CCMP2878**	**30806**	**5953**	**193.884**	**49.11**	**Woo et al, 2015**[S2]
**Vitrella brassicaformis (1)**	**CCMP3155**	**23503**	**1064**	**72.700**	**58.09**	**Woo et al, 2015**[S2]
**Gregarina niphandrodes (1)**	**Unknown**	**6606**	**468**	**14.008**	**53.78**	**x**
Cyclospora cayetanensis	CHN_HEN01	7592	2297	44.034	51.84	Liu et al, 2016[S9]
Cystoisospora suis	WienI	11767	7880	81.642	49.32	Palmieri et al, 2017[S10]
Eimeria falciformis	BayerHaberkorn1970	6037	753	43.672	49.86	Heitlinger et al, 2014[S11]
Eimeria tenella	Houghton	8634	4664	51.859	51.33	Reid et al, 2014[S12]
Hammondia hammondi	HH34	8177	3676	64.338	52.83	Walzer et al, 2013[S13]
Neospora caninum	LIV	7266	66	59.103	54.82	Reid et al, 2012[S14]
Sarcocystis neurona	SN3	7089	873	124.411	51.41	Blazejewski et al, 2015[S15]
**Toxoplasma gondii (1)**	**ME49**	**8920**	**2075**	**65.590**	**52.30**	**Lorenzi et al, 2016**[S16]
Babesia bovis	T2Bo	3781	14	8.179	41.59	Brayton et al, 2007[S17]
Babesia microti	RI	3685	6	6.434	36.17	Cornillot et al, 2012[S18]
Babesia ovata	Miyake	5108	91	14.453	49.27	Yamagishi et al, 2017[S19]
Theileria equi	WA	5397	12	11.674	39.48	Kappmeyer et al, 2012[S20]
Theileria orientalis	Shintoku	4058	6	9.010	41.55	Hayashida et al, 2012[S21]
Theileria parva	Muguga	4167	10	8.353	34.04	Gardner et al, 2005[S22]
Cytauxzoon felis	Winnie	4389	357	9.108	31.81	Tarigo et al, 2013[S23]
Plasmodium berghei	ANKA	5245	21	18.780	22,04	Otto et al, 2014[S24]
**Plasmodium falciparum (1, 2)**	**3D7**	**5712**	**16**	**23.332**	**19.34**	**Gardner et al, 2002**[S25]
Plasmodium vivax	P01	6830	242	29.052	39.78	Auburn et al, 2016[S26]
Plasmodium reichenowi (2)	G01	5909	48	24.471	24.47	Otto et al, 2014[S24]

(1) subset of 6 species (4 apicomplexan + 2 chromerids) used in some comparative analyses, including search for orthogroups and genomic metrics
(2) species used to date the divergence of *P.* cf. *gigantea* A and B
(a) data from VEupathDB release 41[S27]
(b) data obtained with QUAST[S28]

Table S1. Metrics of 25 apicomplexan and chromerids genomes, considered representative for comparative analyses. Related to Table 1 and Figure 2.

Lobster Specimen	Sampling date	Host from Tanks/Bay	Host sex	Host Lenght (cm)	Host Weight (g)	Cysts load in host rectal ampulla	Trophozoites Load in host gut lumen
#1	24/05/2016	Tanks	male	25	355	< 10	none
#2	24/05/2016	Tanks	male	29	645	10-100	none
#3	24/05/2016	Tanks	female	26	450	10-100	none
#4	25/05/2016	Tanks	female	29	620	< 10	none
#5	25/05/2016	Tanks	male	25	420	10-100	none
#6	26/05/2016	Tanks	male	29	745	< 10	< 10
#7	26/05/2016	Tanks	male	25	375	10-100	< 10
#8	27/05/2016	Tanks	female	27	445	10-100	none
#9	27/05/2016	Tanks	male	26	490	10-100	none
#10	30/05/2016	Tanks	male	26	470	none	none
#11	30/05/2016	Bay	female	25	420	10-100	none
#12	31/05/2016	Bay	male	24	465	100-1000	>10
#13	31/05/2016	Bay	female	24	435	100-1000	none
#14	18/10/2016	Tanks	male	27	485	~200	< 10
#15	19/10/2016	Tanks	male	26	685	none	none
#16	19/10/2016	Tanks	female	27	535	none	none
#17	20/10/2016	Bay	male	23	455	100-300	< 10
#18	20/10/2016	Bay	male	25	450	10-100	< 10
#19	24/10/2016	Bay	female	25	510	10-100	none
#20	24/10/2016	Tanks	male	23	405	10-100	>10
#21	25/10/2016	Tanks	male	27	550	none	none
#22	26/10/2016	Tanks	female	30	895	10-100	>10
#23	26/10/2016	Tanks	male	29	510	10-100	none
#24	03/10/2017	Tanks	female	27	505	10-100	>10
#25	03/10/2017	Tanks	female	28	580	10-100	>10
#26	04/10/2017	Bay	male	34	815	10-100	none
#27	05/10/2017	Bay	male	26	515	100-500	>200
#28	06/10/2017	Tanks	female	30	635	< 10	none
#29	06/10/2017	Tanks	male	26	560	10-100	none
#30	09/10/2017	Tanks	male	27	655	none	none
#31	09/10/2017	Tanks	male	28	710	none	none
#32	09/10/2017	Tanks	female	26	450	10-100	none
#33	11/10/2017	Tanks	male	26	470	10-100	>10
#34	12/10/2017	Tanks	male	27	510	10-100	none
#35	17/07/2015	Bay	ND	ND	ND	100-300	none

Table S2. Sampling of the lobster specimen. Related to Figure 1.

Trophozoite specimen	Host specimen (origin)	Length (µm)	Width ± SD (µm) (n=number of measures)
#1	H0 (Bay)	1796	32.8±4.5 (n=13)
#2	H0 (Bay)	>983	34.2±3.9 (n=14)
#3	H12 (Bay)	none	45.2±3.6 (n=3)
#4	H6 (Tank)	1424	51.5±8.4 (n=25)
#5	H12 (Bay)	none	66 to 23µm
#6	H12 (Bay)	none	none
#7	H12 (Bay)	>1043	71.5±10.1
#8	H12 (Bay)	1858	43.3±7.7 (n=13)
#9	H12 (Bay)	2585	55.5±4.5 (n=6)
#10	H12 (Bay)	none	41
#11	H20 (Tank)	2000	36.2±3.1 (n=6)
#12	H20 (Tank)	2177	37.6±7.3 (n=6)
#13	H20 (Tank)	2222	30.6±1.9 (n=6)
#14	H20 (Tank)	>1062	51.0±6.6 (n=6)
#15	H20 (Tank)	>681	31.5±1.9 (n=6)
**Mean value**			**41.8±10.4 (n=104)**

(a) mean values for 15 trophozoites from indicated hosts specimen
(b) The sign > corresponds to truncated trophozoites that could not be measured in full.

Table S3. Length and width of trophozoites, related to Figure 1. All values are based on SEM images.

Cyst specimen	Host specimen (origin)	Diameter (µm) (a)
#1	H#12 (Bay)	118.7±4.5 (n=8) *
#2	H#6 (Tank)	168.4±9.1 (n=3) *
#3	H#12 (Bay)	135.3±1.6 (n=4)
#4	H#12 (Bay)	168.6±2.5 (n=4)
#5	H#12 (Bay)	157.1±5.4 (n=4)
#6	H#12 (Bay)	122.7±2.8 (n=4)
#7	H#12 (Bay)	162.0±4.0 (n=4)
#8	H#12 (Bay)	120.6±3.6 (n=4)
#9	H#4 (Tank)	137.0±1.7 (n=4)
#10	H#4 (Tank)	108.4±10.6 (n=4)
#11	H#4 (Tank)	109.8±3.9 (n=4) *
#12	H#4 (Tank)	168.6x128.4 (oval) (n=2)
#13	H#4 (Tank)	220.6±7.0 (n=4)
#14	H#4 (Tank)	252.2±3.7 (n=4)
#15	H#4 (Tank)	240.9±6.5 (n=4)
#16	H#4 (Tank)	211.0±10.9 (n=4)
#17	H#4 (Tank)	141.9±2.0 (n=4)
#18	H#4 (Tank)	118.1±1.4 (n=4)
#19	H#4 (Tank)	104.6±3.2 (n=4)
#20	H#4 (Tank)	108.3±3.1 (n=4)
#21	H#4 (Tank)	121.9±4.1 (n=4)
#22	H#4 (Tank)	129.7±6.1 (n=4)
#23	H#4 (Tank)	124.7±3.6 (n=4)
#24	H#4 (Tank)	230±9.9 (n=4)
#25	H#4 (Tank)	220.6±7.0 (n=4)
**Mean**		**151.1±45.3 (n=97)**

(a) Mean values measured for 25 cysts. One diameter ± standard deviation (for spherical cysts) or two measures (for oval cyst #12) are given. n, number of measures.
* cysts that were further investigated for gymnospores and zoites measures (see Table S6).

Table S4. Diameters of cysts, related to Figure 1. All values are based on SEM images.

**Origin of gymnospores and zoites**	**Host specimen (origin)**	**Gymnospore Diameter (µm)(a)**	**Zoite length (µm) (a)**	**Zoite width (µm) (a)**
Cyst#1	H#12 (Bay)	4.97±0.37 (n=60)	1.17±0.07 (n=7)	0.565±0.218 (n=50)
Cyst#2	H#6 (Tanks)	5.79±0.62 (n=97)	1.04±0.11 (n=45)	0.616±0.033 (n=11)
Cyst#11	H#4 (Tanks)	6.04±0.72 (n=56)	1.09±0.07 (n=16)	0.674±0.043 (n=35)
JS-463b_0003	H#4 (Tanks)			0.660±0.045 (n=10)
JS-463b_0016 (a)	H#4 (Tanks)	5.30±0.05 (n=4)		0.643±0.042 (n=10)
JS-463b_0016 (b)	H#4 (Tanks)	5.08±0.16 (n=4)		
JS-463b_0016 (c)	H#4 (Tanks)	5.92±0.18 (n=4)	1.19±0.09 (n=3)	0.673±0.086 (n=3)
JS-463b_0020 (a)	H#4 (Tanks)	5.26±0.18 (n=4)		0.662±0.035 (n=10)
JS-463b_0020 (b)	H#4 (Tanks)	5.33±0.04 (n=4)		
JS-463b_0020 (c)	H#4 (Tanks)	6.21±0.22 (n=4)		
JS-463b_0020 (d)	H#4 (Tanks)	6.63±0.30 (n=4)		
JS-463b_0020 (e)	H#4 (Tanks)	6.24±0.18 (n=4)		
JS-463b_0020 (f)	H#4 (Tanks)	5.64±0.11 (n=4)		
JS-463b_0027 (a)	H#4 (Tanks)	5.66±0.19 (n=4)		0.646±0.050 (n=10)
JS-463b_0027 (b)	H#4 (Tanks)	4.69±0.10 (n=4)		0.656±0.029 (n=10)
JS-463b_0027 (c)	H#4 (Tanks)	5.70±0.10 (n=4)		0.643±0.039 (n=10)
JS-463b_0028	H#4 (Tanks)		1.09±0.09 (n=10)	
JS-463b_0030	H#4 (Tanks)	5.80±0.08 (n=4)		0.684±0.085 (n=10)
JS-463b_0036	H#4 (Tanks)		1.16±0.07 (n=13)	0.613±0.037 (n=10)
**Mean**		**5.63±0.69 (n=265)**	**1.04±0.16 (n=105)**	**0.630±0129 (n=176)**

(a) n, number of measures.

Table S5. Diameters of gymnospores and zoites, related to Figure 1. Diameters were measured for hundreds of gymnospores within cysts (3 first lines) or released from cysts (remaining lines). Whenever possible, length and apical width ± standard deviation of their constitutive zoites were also measured. All values are based on SEM images.

Video record	Length of recording (s)	Trophozoites number	Length (µm)	Speed (µm/s)
G5310002	40	T10<br>T11<br>T12<br>T13<br>T14<br>T15	~2190<br>~1876<br>~2113<br>~2113<br>~1801<br>~1807	51.6<br>48.9<br>50.3<br>51.8<br>49.8<br>51.4
G5310003	34	T3<br>T4<br>T5	~3100<br>~4500<br>~3900	87-89<br>100-109<br>108-115
G5310004	60 and 48	T1<br>T2	~3000<br>~3600	56-63<br>80-81
G5310018	20	T6<br>T7<br>T8<br>T9	~2540<br>~4600<br>~4100<br>~3595	76<br>97-103<br>104<br>91-94

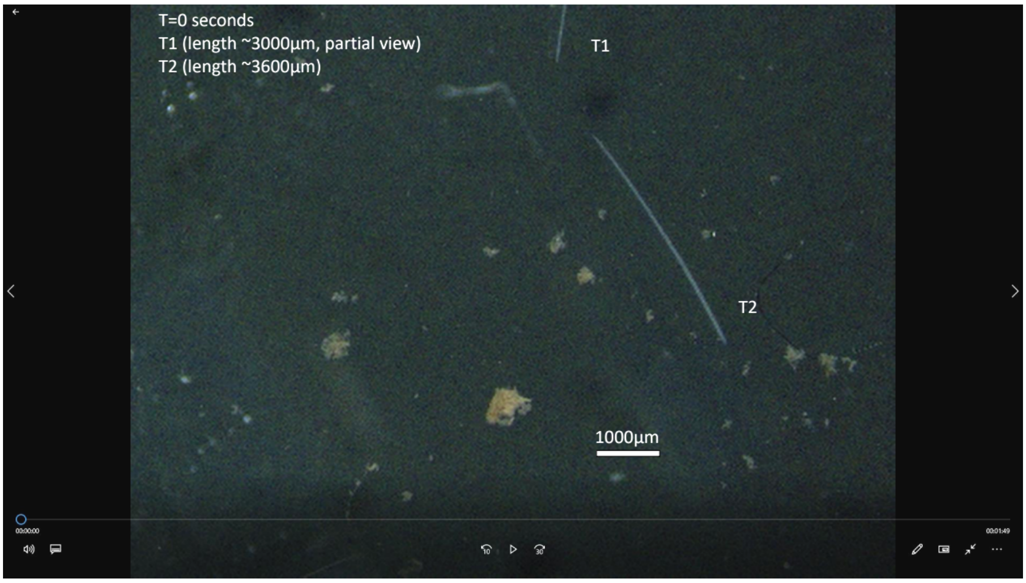

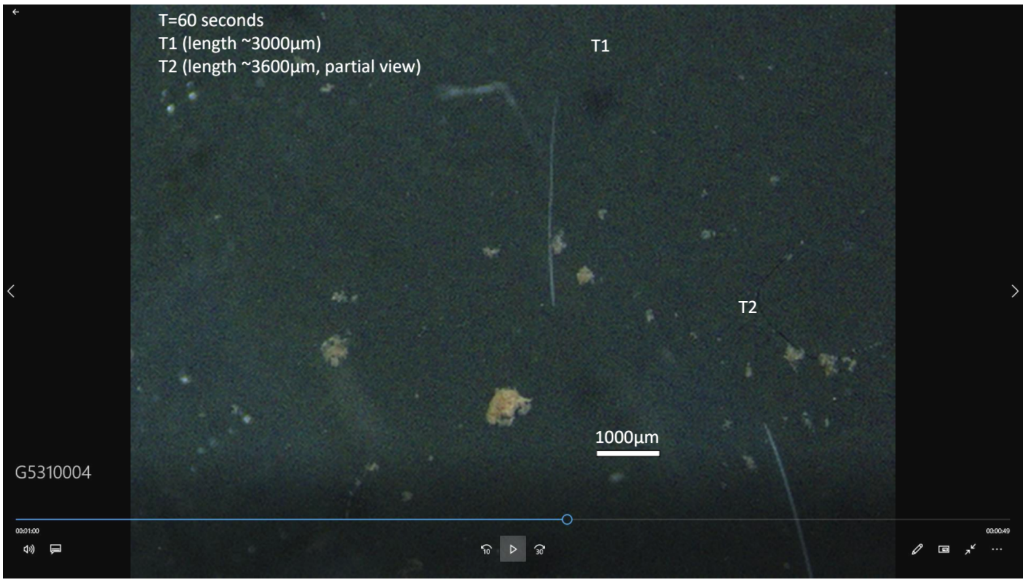

Table S6. Gliding recordings, related to Figure 1 and Film S1. All recordings are from trophozoites collected from Lobsters#12 and #13 on 31/05/2016. Due to the lack of scale on these videos, we used the mean width of trophozoites, as determined by using SEM images (41.8±10.4µm, see Table S4), to calibrate the other measures. Two screenshots (t=0 and t=60') from the supplemental film are reproduced.

Primer name	Primer sequence	orientation	reference
LWA1	5’- GGAAGGCAGCAGGCGCGC - 3’	forward	Schrevel et al., 2016[S29]
EukP3	5’- GACGGGCGGTGTGTAC - 3’	reverse	Lara et al., 2007[S30]
28d5	5’- CCGCTAAGGAGTGTGTAACAAC - 3’	forward	Simdyanov et al., 2015[S1]
28r3.2	5’- ACTCCTYRGTCCGTGTTTCA - 3’	reverse	Simdyanov et al., 2015[S1]
28r2	5’- TACTTGTYBRCTATCG - 3’	reverse	Simdyanov et al., 2015[S1]
d6new	5’- GGTGGTGCATGGCCAAACTT - 3’	forward	Modified from Simdyanov et al., 2015[S1]
28d5short	5’- GCTAAGGAGTGTGTAACAAC - 3’	forward	Modified from Simdyanov et al., 2015[S1]
28r7new	5’- TAATTTGCCGACTTCCCTCA - 3’	reverse	Modified from Simdyanov et al., 2015[S1]
PIF5F	5’- ACATTCCTTGGGTTACCC - 3’	forward	This study
PIF6F	5’- TAACGACCCGAAAATCGG - 3’	forward	This study
PIF7F	5’- CATGCTAACACAAGGGGG - 3’	forward	This study
PIF8F	5’- CCGACAGTTTAACTAAAACC - 3’	forward	This study
PIF9F	5’- GAGATCATATCGACGCGG- 3’	forward	This study
PIF5R	5’- CATCAGTGCGACGATACC - 3’	reverse	This study
PIF6R	5’- GTTTGAGAATCAGTCGAGG - 3’	reverse	This study
PIF7R	5’- CTTTCGACTTCCGACAGC - 3’	reverse	This study
PIF8R	5’- TTGTTTGCTATCGGTATAGG - 3’	reverse	This study
PIF9R	5’- AAATCTCAAGAGAGATGGAG- 3’	reverse	This study
PIF10R	5’- GCTAAGGATCGATAGGCC - 3’	reverse	This study

Table S7. List of primers used for ribosomal locus amplification and sequencing by Sanger technology. Related to Figure S6.

**Protein name**	***P. cf. gigantea* A**	***P. cf. gigantea* B**
Actin	KAH0480873.1<br>KAH0486428.1<br>KAH0477721.1<br>KAH0476702-3.1	KAH0488956.1<br>KAH0472333.1<br>KAH0482523.1
Profilin	KAH0486108.1	KAH0488334.1
Formin	KAH0487606.1<br>KAH0473072-3.1	KAH0488107-8.1<br>KAH0473233-4.1
ADF_cofilin	KAH0487744.1	KAH0488925.1
CAP	KAH0483375-6.1	KAH0475361-2.1
Cpβ F-actin capping protein β-subunit	KAH0474592:4.1	KAH0471516:8.1
MyosinACDE ClassXIV	KAH0483751.1<br>KAH0483531.1<br>KAH0484363.1<br>KAH0481456.1<br>KAH0475489.1	KAH0487710:12.1<br>KAH0480515.1<br>KAH0484061.1<br>KAH0483307:11.1
MyosinH ClassXIV	KAH0473906:8.1	KAH0486610:12.1
MTIP_MLC1	KAH0476563.1	KAH0475263.1
GAP40	KAH0477413-4.1	KAH0478356.1
GAP45 (partial 3')	KAH0473219.1	KAH0477289.1
GAPM1	GAPM3 KAH0485741.1<br>GAPMx KAH0485173.1<br>GAPMx KAH0472651.1	GAPM3 KAH0485644.1<br>GAPMx KAH0481982.1
GAC	KAH0484909-10.1	KAH0477618:20.1
ROM4	KAH0485928-9.1<br>KAH0475712:14.1	KAH0480431:33.1<br>KAH0472445-6.1
AKMT	KAH0472731.1	KAH0488385.1
CDPK1(Tg)/CDPK4(Pf)	KAH0477425:27.1	KAH0474276:78.1
CDPK3(Tg)/CDPK1(Pf)	KAH0482406.1	KAH0483722:23.1
CDPK5(Pf)/CDPK5(Tg)	KAH0475451:53.1	KAH0475693:95.1
DGK1	KAH0473632-3.1	KAH0476753.1
DOC2.1	KAH0486912:14.1	KAH0488625:27.1
TSP-1 (a)	KAH0483741:43.1	KAH0487684-5.1
TSP-2 (a)	KAH0474072.1	KAH0482614-5.1
TSP2 (a)	KAH0472958:61.1	KAH0473100:103.1<br>KAH0473117.1
TSP_EGF-1 (a)	KAH0477971:73.1	KAH0472910:12.1
TSP_EGF-2 (a)	KAH0483270-1.1	KAH0483538:40.1

(a) TRAP like candidates

Table S8. *P. cf. gigantea* A and B glideosome and TRAP-like proteins identifiers, related to Figure 5.

Supplemental References
S1.
Simdyanov, T.G., Diakin, A.Y., and Aleoshin, V.V. (2015). Ultrastructure and 28S rDNA Phylogeny of Two Gregarines: *Cephaloidophora cf. communis* and *Heliospora cf. longissima* with Remarks on Gregarine Morphology and Phylogenetic Analysis. Acta Protozoologica 54, 241–262. 10.4467/16890027AP.15.020.3217.
S2.
Woo, Y.H., Ansari, H., Otto, T.D., Klinger, C.M., Kolisko, M., Michálek, J., Saxena, A., Shanmugam, D., Tayyrov, A., Veluchamy, A., et al. (2015). Chromerid genomes reveal the evolutionary path from photosynthetic algae to obligate intracellular parasites. ELife 4. 10.7554/eLife.06974.
S3.
Mathur, V., Kolísko, M., Hehenberger, E., Irwin, N.A.T., Leander, B.S., Kristmundsson, Á., Freeman, M.A., and Keeling, P.J. (2019). Multiple Independent Origins of Apicomplexan-Like Parasites. Current Biology 29, 2936-2941.e5. 10.1016/j.cub.2019.07.019.
S4.
Janouškovec, J., Paskerova, G.G., Miroliubova, T.S., Mikhailov, K.V., Birley, T., Aleoshin, V.V., and Simdyanov, T.G. (2019). Apicomplexan-like parasites are polyphyletic and widely but selectively dependent on cryptic plastid organelles. eLife 8, e49662. 10.7554/eLife.49662.
S5.
Mathur, V., Kwong, W.K., Husnik, F., Irwin, N.A.T., Kristmundsson, Á., Gestal, C., Freeman, M., and Keeling, P.J. (2021). Phylogenomics Identifies a New Major Subgroup of Apicomplexans, Marosporida *class nov.*, with Extreme Apicoplast Genome Reduction. Genome Biology and Evolution 13, evaa244. 10.1093/gbe/evaa244.
S6.
Guo, Y., Tang, K., Rowe, L.A., Li, N., Roellig, D.M., Knipe, K., Frace, M., Yang, C., Feng, Y., and Xiao, L. (2015). Comparative genomic analysis reveals occurrence of genetic recombination in virulent *Cryptosporidium hominis* subtypes and telomeric gene duplications in *Cryptosporidium parvum*. BMC Genomics 16. 10.1186/s12864-015-1517-1.
S7.
Ifeonu, O.O., Chibucos, M.C., Orvis, J., Su, Q., Elwin, K., Guo, F., Zhang, H., Xiao, L., Sun, M., Chalmers, R.M., et al. (2016). Annotated draft genome sequences of three species of *Cryptosporidium* : *Cryptosporidium meleagridis* isolate UKMEL1, *C. baileyi* isolate TAMU-09Q1 and *C. hominis* isolates TU502_2012 and UKH1. Pathogens and Disease 74, ftw080. 10.1093/femspd/ftw080.
S8.
Abrahamsen, M.S., Templeton, T.J., Enomoto, S., Abrahante, J.E., Zhu, G., Lancto, C.A., Deng, M., Liu, C., Widmer, G., Tzipori, S., et al. (2004). Complete Genome Sequence of the Apicomplexan *Cryptosporidium parvum*. 304, 6.
S9.
Liu, S., Wang, L., Zheng, H., Xu, Z., Roellig, D.M., Li, N., Frace, M.A., Tang, K., Arrowood, M.J., Moss, D.M., et al. (2016). Comparative genomics reveals *Cyclospora cayetanensis* possesses coccidia-like metabolism and invasion components but unique surface antigens. BMC Genomics 17, 316. 10.1186/s12864-016-2632-3.
S10.
Palmieri, N., Shrestha, A., Ruttkowski, B., Beck, T., Vogl, C., Tomley, F., Blake, D.P., and Joachim, A. (2017). The genome of the protozoan parasite *Cystoisospora suis* and a reverse vaccinology approach to identify vaccine candidates. International Journal for Parasitology 47, 189–202. 10.1016/j.ijpara.2016.11.007.
S11.
Heitlinger, E., Spork, S., Lucius, R., and Dieterich, C. (2014). The genome of *Eimeria falciformis*, reduction and specialization in a single host apicomplexan parasite. BMC Genomics 15, 696. 10.1186/1471-2164-15-696.
S12.
Reid, A.J., Blake, D.P., Ansari, H.R., Billington, K., Browne, H.P., Bryant, J., Dunn, M., Hung, S.S., Kawahara, F., Miranda-Saavedra, D., et al. (2014). Genomic analysis of the causative agents of coccidiosis in domestic chickens. Genome Res. 24, 1676–1685. 10.1101/gr.168955.113.

S13.
Walzer, K.A., Adomako-Ankomah, Y., Dam, R.A., Herrmann, D.C., Schares, G., Dubey, J.P., and Boyle, J.P. (2013). Hammondia hammondi, an avirulent relative of *Toxoplasma gondii*, has functional orthologs of known *T. gondii* virulence genes. Proceedings of the National Academy of Sciences 110, 7446–7451. 10.1073/pnas.1304322110.
S14.
Reid, A.J., Vermont, S.J., Cotton, J.A., Harris, D., Hill-Cawthorne, G.A., Könen-Waisman, S., Latham, S.M., Mourier, T., Norton, R., Quail, M.A., et al. (2012). Comparative Genomics of the Apicomplexan Parasites *Toxoplasma gondii* and *Neospora caninum*: Coccidia Differing in Host Range and Transmission Strategy. PLoS Pathogens 8, e1002567. 10.1371/journal.ppat.1002567.
S15.
Blazejewski, T., Nursimulu, N., Pszenny, V., Dangoudoubiyam, S., Namasivayam, S., Chiasson, M.A., Chessman, K., Tonkin, M., Swapna, L.S., Hung, S.S., et al. (2015). Systems-Based Analysis of the *Sarcocystis neurona* Genome Identifies Pathways That Contribute to a Heteroxenous Life Cycle. mBio 6, e02445-14. 10.1128/mBio.02445-14.
S16.
Lorenzi, H., Khan, A., Behnke, M.S., Namasivayam, S., Swapna, L.S., Hadjithomas, M., Karamycheva, S., Pinney, D., Brunk, B.P., Ajioka, J.W., et al. (2016). Local admixture of amplified and diversified secreted pathogenesis determinants shapes mosaic *Toxoplasma gondii* genomes. Nat Commun 7, 10147. 10.1038/ncomms10147.
S17.
Brayton, K.A., Lau, A.O.T., Herndon, D.R., Hannick, L., Kappmeyer, L.S., Berens, S.J., Bidwell, S.L., Brown, W.C., Crabtree, J., Fadrosh, D., et al. (2007). Genome Sequence of *Babesia bovis* and Comparative Analysis of Apicomplexan Hemoprotozoa. PLoS Pathogens 3, e148. 10.1371/journal.ppat.0030148.
S18.
Cornillot, E., Hadj-Kaddour, K., Dassouli, A., Noel, B., Ranwez, V., Vacherie, B., Augagneur, Y., Brès, V., Duclos, A., Randazzo, S., et al. (2012). Sequencing of the smallest Apicomplexan genome from the human pathogen *Babesia microti*. Nucleic Acids Research 40, 9102–9114. 10.1093/nar/gks700.
S19.
Yamagishi, J., Asada, M., Hakimi, H., Tanaka, T.Q., Sugimoto, C., and Kawazu, S. (2017). Whole-genome assembly of *Babesia ovata* and comparative genomics between closely related pathogens. BMC Genomics 18. 10.1186/s12864-017-4230-4.
S20.
Kappmeyer, L.S., Thiagarajan, M., Herndon, D.R., Ramsay, J.D., Caler, E., Djikeng, A., Gillespie, J.J., Lau, A.O., Roalson, E.H., Silva, J.C., et al. (2012). Comparative genomic analysis and phylogenetic position of *Theileria equi*. BMC Genomics 13, 603. 10.1186/1471-2164-13-603.
S21.
Hayashida, K., Hara, Y., Abe, T., Yamasaki, C., Toyoda, A., Kosuge, T., Suzuki, Y., Sato, Y., Kawashima, S., Katayama, T., et al. (2012). Comparative Genome Analysis of Three Eukaryotic Parasites with Differing Abilities To Transform Leukocytes Reveals Key Mediators of *Theileria*-Induced Leukocyte Transformation. mBio 3. 10.1128/mBio.00204-12.
S22.
Gardner, M.J. (2005). Genome Sequence of *Theileria parva*, a Bovine Pathogen That Transforms Lymphocytes. Science 309, 134–137. 10.1126/science.1110439.
S23.
Tarigo, J.L., Scholl, E.H., Bird, D.McK., Brown, C.C., Cohn, L.A., Dean, G.A., Levy, M.G., Doolan, D.L., Trieu, A., Nordone, S.K., et al. (2013). A Novel Candidate Vaccine for Cytauxzoonosis Inferred from Comparative Apicomplexan Genomics. PLoS ONE 8, e71233. 10.1371/journal.pone.0071233.
S24.
Otto, T.D., Böhme, U., Jackson, A.P., Hunt, M., Franke-Fayard, B., Hoeijmakers, W.A.M., Religa, A.A., Robertson, L., Sanders, M., Ogun, S.A., et al. (2014). A comprehensive evaluation of rodent malaria parasite genomes and gene expression. BMC Biology 12. 10.1186/s12915-014-0086-0.

S25.
Gardner, M.J., Hall, N., Fung, E., White, O., Berriman, M., Hyman, R.W., Carlton, J.M., Pain, A., Nelson, K.E., Bowman, S., et al. (2002). Genome sequence of the human malaria parasite *Plasmodium falciparum*. Nature 419, 498–511. 10.1038/nature01097.
S26.
Auburn, S., Böhme, U., Steinbiss, S., Trimarsanto, H., Hostetler, J., Sanders, M., Gao, Q., Nosten, F., Newbold, C.I., Berriman, M., et al. (2016). A new *Plasmodium vivax* reference sequence with improved assembly of the subtelomeres reveals an abundance of pir genes. Wellcome Open Research 1, 4. 10.12688/wellcomeopenres.9876.1.
S27.
Aurrecoechea, C., Barreto, A., Basenko, E.Y., Brestelli, J., Brunk, B.P., Cade, S., Crouch, K., Doherty, R., Falke, D., Fischer, S., et al. (2017). EuPathDB the eukaryotic pathogen genomics database resource. Nucleic Acids Res 45, D581–D591. 10.1093/nar/gkw1105.
S28.
Gurevich, A., Saveliev, V., Vyahhi, N., and Tesler, G. (2013). QUAST: quality assessment tool for genome assemblies. Bioinformatics 29, 1072–1075. 10.1093/bioinformatics/btt086.
S29.
Schrével, J., Valigurová, A., Prensier, G., Chambouvet, A., Florent, I., and Guillou, L. (2016). Ultrastructure of *Selenidium pendula*, the Type Species of Archigregarines, and Phylogenetic Relations to Other Marine Apicomplexa. Protist 167, 339–368. 10.1016/j.protis.2016.06.001.
S30.
Lara, E., Berney, C., Ekelund, F., Harms, H., and Chatzinotas, A. (2007). Molecular comparison of cultivable protozoa from a pristine and a polycyclic aromatic hydrocarbon polluted site. Soil Biology and Biochemistry 39, 139–148. 10.1016/j.soilbio.2006.06.017.
